# A two-pronged approach against glioblastoma: drug repurposing and nanoformulation design for in situ-controlled release

**DOI:** 10.1007/s13346-023-01379-8

**Published:** 2023-08-13

**Authors:** Maria Mendes, Francisco Branco, Rui Vitorino, João Sousa, Alberto Pais, Carla Vitorino

**Affiliations:** 1 Faculty of Pharmacy, University of Coimbra, Azinhaga de Santa Comba, Pólo das Ciências da Saúde, 3000-548 Coimbra, Portugal; 2 Coimbra Chemistry Centre, Institute of Molecular Sciences - IMS, Department of Chemistry, University of Coimbra, 3004-535 Coimbra, Portugal; 3 iBiMED-Department of Medical Sciences, University of Aveiro, Aveiro, Portugal; 4 Department of Surgery and Physiology, Faculty of Medicine, UnIC, University of Porto, Porto, Portugal; 5 LAQV-REQUIMTE, Chemistry Department, University of Aveiro, Aveiro, Portugal

**Keywords:** Drug repurposing, Bioinformatics, Ultra-small nanostructured lipid carriers, Thermoresponsive matrix, In situ controlled release

## Abstract

Glioblastoma (GB) is one of the most lethal types of neoplasms. Its biologically aggressive nature and the presence of the blood–brain barrier (BBB) limit the efficacy of standard therapies. Several strategies are currently being developed to both overcome the BBB and deliver drugs site specifically to tumor cells. This work hypothesizes a two-pronged approach to tackle GB: drug repurposing with celecoxib (CXB) and a nanoformulation using ultra-small nanostructured lipid carriers (usNLCs). CXB antitumor druggable activity was inspected bioinformatically and screened in four glioma cell lines aiming at the comparison with temozolomide (TMZ), as standard of care. Delving into formulation design, it was tailored aiming at (i) improving the drug solubility/loading properties, (ii) assigning a thermal-triggerable drug release based on a lipid matrix with a low melting point, and (iii) enhancing the cytotoxic effect by selecting a template targetable to tumor cells. For this purpose, an integrated analysis of the critical material attributes (CMAs), critical process parameters (CPPs), and critical quality attributes (CQAs) was conducted under the umbrella of a quality by design approach. CMAs that demonstrate a high-risk level for the final quality and performance of the usNLCs include the drug solubility in lipids (solid and liquid), the lipid composition (envisioning a thermoresponsive approach), the ratio between lipids (solid vs. liquid), and the surfactant type and concentration. Particle size was shown to be governed by the interaction lipid-surfactant followed by surfactant type. The drug encapsulation did not influence colloidal characteristics, making it a promising carrier for lipophilic drugs. In general, usNLCs exhibited a controlled drug release during the 72 h at 37 °C with a final release of ca. 25%, while at 45 °C this was doubled. The in vitro cellular performance depended on the surfactant type and lipid composition, with the formulations containing a sole solid lipid (Suppocire^®^ NB) and Kolliphor^®^ RH40 as surfactant being the most cytotoxic. usNLCs with an average diameter of ca. 70 nm and a narrow size distribution (PdI lower than 0.2) were yielded, exhibiting high stability, drug protection, sustained and thermo-sensitive release properties, and high cytotoxicity to glioma cells, meeting the suitable CQAs for parenteral administration. This formulation may pave the way to a multi-addressable purpose to improve GB treatment.

**Graphical Abstract:**

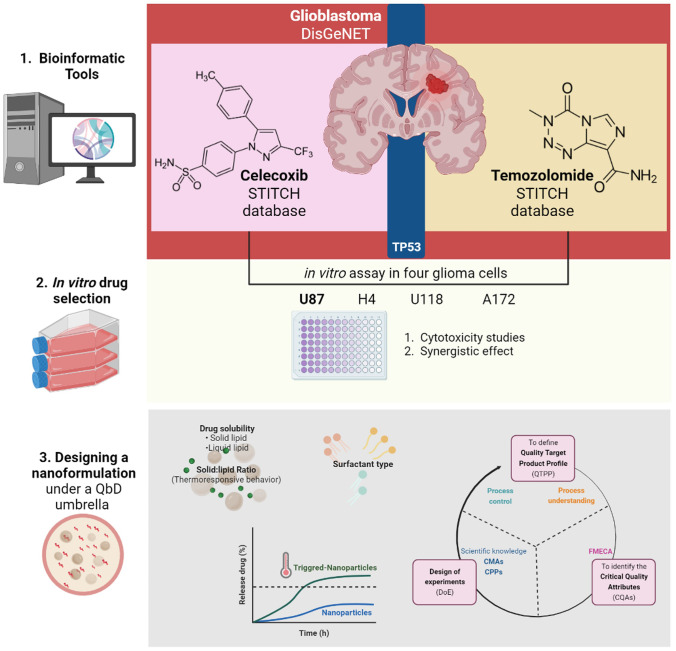

## Introduction

Glioblastoma (GB) is one of the most morbid and lethal types of neoplasms, with unique anatomic, physiologic, and pathologic features that persist after treatment with standard therapies, such as surgery followed by radiotherapy with concomitant and adjuvant chemotherapy with TMZ [1–3]. The biological aggressiveness of the tumor and the presence of the blood–brain barrier (BBB) are limiting factors for its effective treatment. The need for new drugs and/or therapeutic strategies for GB is clear and urgent. Several strategies are being developed to tackle this issue, including the two-pronged approach addressed in this work: drug repurposing and formulation design tailored to cross the dual challenge BBB-blood–brain tumor barrier.

Drug repurposing circumvents the limitations associated with the approval of new drugs, such as the long development process of novel molecules and the associated costs. The advantages of drug repurposing lie mainly in the knowledge of the mechanisms of action, molecular targets, well-established pharmacological properties, and targeting of different signaling pathways or receptors. Celecoxib (CXB), a selective cyclooxygenase-2 (COX-2) inhibitor, has been reported to mediate growth inhibitory effects, induce apoptosis, and reduce the risk for the occurrence and progression of several cancers, including GB [4–6]. Indeed, high levels of COX-2 are present in human malignant glioma cells and COX-2 play an important role in glioma resistance and progression [7, 8]. However, long-term intake of CXB could be toxic to the gastrointestinal and cardiovascular systems due to impairment of prostaglandin synthesis, resulting in side effects for both systems. The severity of these side effects increases with high doses, considering the physicochemical properties of CXB [4]. Therefore, the development of nanoparticles for CXB delivery is pointed out as a technological strategy [7, 9].

Nanoparticles (NPs) are widely used to improve the physicochemical properties of the drugs while enabling the encapsulation of a large amount of one or more drugs. NP benefits rely on colloidal properties, including small size and narrow size distributions, and surface properties tailored to bypass biological barriers and increase drug accumulation in the tumor tissue. Surface modification can pursue the enhancement of therapeutic effect, for example, by coupling therapeutic moieties or targeting molecules to control the fate of NPs in vivo. Among the various types of NPs, solid lipid-matrix NPs arise as promising candidates for BBB-cross ability and GB treatment [10–13]. Nanostructured lipid carriers (NLCs), the second generation of solid lipid-matrix NPs, have been used due to their advantages over other NPs, including (i) small size and large specific surface area; (ii) high drug loading, particularly for poorly water-soluble compounds; (iii) drug protection and controlled release promoted by their solid nature; (iv) biocompatibility and biodegradability stemming from the lipids employed; (v) highly scalable capacity; (vi) versatility in composition, depending on the affinity of the drug(s) for the lipids; and (vii) surface functionalization to enhance their therapeutic activity [14–16]. More recently, another form of solid lipid matrix NPs has been developed, termed ultra-small nanostructured lipid carriers (usNLCs), which combine all the previously described advantages with a higher liquid:solid lipid ratio, and consequently higher drug loading, and a size below 100 nm [17, 18]. The particle size below 100 nm assumes particular relevance in terms of BBB cross ability and enhanced permeability and retention in the tumor tissue. The lipid matrix assumes a critical importance in the design of these nanoconstructs, not only to increase the amount of entrapped drug but also to modulate its release and even make it responsive to several stimuli, including temperature, e.g., by the thorough selection of low-melting-point solid lipids [19–21]. The development of usNLCs sensitive to temperature enables a combination between chemotherapy and hyperthermia, taking advantage of the synergistic effect between both therapeutic strategies. Hyperthermia, a process in which body tissues are exposed to high temperatures (up to 39–45 °C), is sparking particular interest due to improvements in cancer treatment in combination with chemotherapeutic agents, e.g., by enhancing drug release [22, 23].

The design and optimization of a usNLC formulation should be based on certain premises, a priori linked to input material properties, and the implementation of the systematic quality by design (QbD) approach would be helpful to achieve the “right composition the first time”. For that, QbD elements must be set forth. This process includes the following steps [24–27]: determining the quality target product profile (QTPP), defining the critical quality attributes (CQAs), and from that, establishing the critical material attributes (CMAs) and the critical process parameters (CPPs) based on prior knowledge when performing risk assessment and conducting design of experiments (DoE) to build a design space and verify its feasibility and robustness.

The QTPP summarizes the product quality characteristics regarding safety and efficacy, considering the drug, dosage form, delivery system, and route of administration, among others. The identification of the CQAs from QTPP is based on the severity of the effects on the in vivo performance of the product. The selection of CMAs, the most impacting variables for CQAs, was carried out through risk analysis. The risk assessment carried out through failure mode, effects, and criticality analysis (FMECA) can help identify the potential risks or failure modes affecting the quality attributes of the usNLCs formulation. As per the preliminary studies, drug solubility in lipids (solid and liquid), lipid composition, ratio between lipids (solid *vs*. liquid), and surfactant type or concentration were established as CMAs. The CPPs, such as high-pressure homogenization (HPH) time and pressure, were kept constant, according to the conditions specified in [28].

In the quest for a repurposing approach, the present work aims at developing and optimizing a CXB-usNLCs formulation with high lipid content and thermoresponsive lipid matrix. The high lipid content (15% w/w) is advantageous because, due to the high drug content, a smaller number of nanoparticles is required to deliver a clinically relevant dose of the therapeutic agent, consequently associated with fewer side effects. On the other hand, by designing a lipid matrix considering solid lipids with a lower melting point (> 39 °C), the systems become sensitive to hyperthermic temperatures by the application of an external field (e.g., laser irradiation, magnetic field), essential for the controlled and targeted release of CXB. The QbD approach was applied to evaluate the best formulation in terms of both colloidal properties and in vitro performance.

## Materials

Polysorbate 80 (Tween^®^ 80), Kolliphor^®^ RH40, IR780, and octadecylamine were provided by Sigma-Aldrich (MO, USA). Oleic acid was acquired from Fluka (Buchs, Switzerland). Labrasol^®^ (caprylocaproyl polyoxyl-8 glycerides), Compritol^®^ 888 ATO (glyceryl dibehenate), Suppocire^®^NB (mono-, di-, and triglyceride esters of C10 to C18 fatty acids, the triester fraction being predominant), Capryol^®^ 90 (propylene glycol monocaprylate), Capryol™ PGMC (propylene glycol monocaprylate-type I), Capmul MCM C8, Labrafil, and Lauroglycol 90 were kindly offered by Gattefossé (Gennevilliers, France). Lipoid S 75^®^ (soy phospholipid) was provided by Lipoid GmbH (Ludwigshafen, Germany). Celecoxib (98.0~102.0% purity) was obtained from Shandong Zhishang Chem Co., Ltd. (Zhangqiu, China). Temozolomide (99%) was purchased from Jinlan Pharm-Drugs Technology Co,. Limited (Hangzhou, China).

Dulbecco’s modified Eagle’s medium (DMEM), fetal bovine serum (FBS), 0.4% trypan blue solution, trypsin–EDTA solution, sodium bicarbonate, phosphate-buffered saline (PBS), penicillin, and streptomycin were obtained from Sigma-Aldrich (MO, USA). Four glioma cell lines were acquired from the American Type Culture Collection (ATCC, Manassas, VA, USA). A172 (ATCC CRL-1620) and H4 (ATCC HTB-148) were used as non-tumorigenic cells, and U118 (ATCC HTB-15) and U87 (ATCC HTB-14) were used as tumorigenic cells.

Ultrapure water (HPLC grade, > 18.2 MΏ) was prepared using a Milli-Q water apparatus (Millipore^®^, USA) and filtered through a 0.22-μm nylon filter before use. All other reagents and solvents were of analytical or high-performance liquid chromatography (HPLC) grade.

## Methods

### Bioinformatics tools

The drug (CXB or TMZ)-gene interactions were obtained from STITCH version 5.0 (http://stitch.embl.de/, accessed on December 7, 2022), which integrates the drug-target knowledge from several sources by exploring the network of chemical relations, along with associated binding proteins [29, 30]. DisGeNET (version 7.0, https://www.disgenet.org/home/, accessed on December 7, 2022) was used to collect the genes and variants associated to GB. This database contains data from scientific literature, and the keywords “Glioblastoma Multiforme,” “Glioblastoma,” “Adult Glioblastoma,” “Recurrent Glioblastoma,” and “Adult Glioblastoma” were used to look up the summary of gene-diseases associations. Significant data were screened with a score_dga_ > 0.11.

The jVenn software (http://jvenn.toulouse.inra.fr/app/index.html, accessed on December 7, 2022) was used to obtain the common targets of CXB, TMZ, and GB by entering the above collected information, from STITCH and DisGeNET [31]. The data was summarized, and the duplicate items were deleted.

UALCAN (http://ualcan.path.uab.edu, accessed on December 7, 2022) was used to provide access to publicly available cancer transcriptome data (The Cancer Genome Atlas, TCGA) [32]. This database was employed to compare expression level of genes between non-tumoral and tumor (in this case GB tissue) samples. Genes whose Kaplan-Meyer curves showed a statistically significant impact on overall survival (OS) were inspected.

### Drug selection: in vitro studies in glioma cells

The cytotoxic effects of CXB and TMZ were tested in four human glioma cell lines: A172, H4, U118, and U87. These cell lines are integrated within the glioma tumor cell panel TCP-1018™ (ATCC) exhibiting varying degrees of genetic complexity and due to genomic mutations in one or more of the following genes according to the Sanger COSMIC database (*CDKN2A, PTEN, and TP53*). Their use was considered to better address tumor heterogeneity.

Cells were cultured in DMEM medium supplemented with 10% heat-inactivated FBS, 1% penicillin–streptomycin, and sodium bicarbonate at 37 °C in a humidified incubator containing 5% CO_2_. Cells were passaged at 70–80% confluence after trypsinization (0.25% trypsin–EDTA in PBS, calcium, and magnesium-free). All assays were performed in triplicate in three independent experiments.

#### Cell viability assay

Glioma cells were seeded in flat-bottom 96-well plates at a density of 20 × 10^4^ cells/well. After 24 h, the medium was replaced with increasing concentrations of working solutions of CXB (7–1700 µM) and TMZ (5–2500 µM) in culture medium with 1% v/v DMSO concentration without compromising cell viability and incubated for 24 and 72 h. The resazurin assay was used to determine the cytotoxicity of drugs. At the end of the experiment, the medium was removed, and 100 μL of 10% (w/V) resazurin solution in DMEM medium was added to the cells and incubated at 37 °C for approximately 2 h. The enzymatic reduction of resazurin to resorufin was determined spectrophotometrically at 570 nm and 600 nm. Cell viability was assessed indirectly according to

$$\%\, \mathrm{Cell\, Viability}=100 \times \frac{{{(Abs}_{570 nm}-{Abs}_{600 nm})}_{Treated \,Cells}}{{{(Abs}_{570 nm}-{Abs}_{600 nm})}_{Control}}$$

A 50% reduction in cell viability (IC_50_) was determined from the concentration–response curves, using Prism version 8.0 (GraphPad Software, Inc., USA) with the sigmoidal curve fitting method.

Co-treatment outcomes were analyzed by calculating the combination index (CI). The IC_50_ values of the two drugs in the U87 cell line were determined. Then, synergistic interaction between TMZ and CXB was analyzed using the CI values calculated on the basis of the Chou and Talalay method [33]. The CI provides a quantitative definition for additive effects (CI = 1), synergism (CI < 1), and antagonist effects (CI > 1) in drug combinations:

$$\mathrm{CI}= \frac{{D}_{1}}{{({D}_{CXB})}_{1} }+\frac{{D}_{2}}{{({D}_{TMZ})}_{2}}$$

*D*_1_ and *D*_2_ are the concentrations of CXB and TMZ used alone that produce 50% cell growth inhibition, whereas ${({D}_{CXB})}_{1}$ and ${({D}_{TMZ})}_{2}$ are the doses of CXB and TMZ used in combination that effectively inhibit 50% of cell growth.

#### Cell apoptosis assay

The cell death mechanism and its percentage induced by celecoxib at the corresponding concentrations of IC_10_, IC_50_, and IC_90_ were measured using the Annexin V-FITC kit as recommended by the manufacturer[34, 35]. At the end of 4 h, cells (4 × 10^4^ cells/well) were harvested and washed with PBS at 4 °C and then resuspended in 100 µL of binding buffer containing 5 µL of annexin V-FITC (AV) and 2 µL of propidium iodide (PI). The plates were protected from light for 15 min at room temperature. The stained cells were analyzed by flow cytometry at an excitation wavelength of 488 nm. Emission fluorescence of AV was recorded in the 530/30 channel, while that of PI was recorded in the 675/30 channel. Cells were gated at acquisition using forward vs. side scatter to eliminate dead cells and debris, and 10,000 gated events were collected for each sample. The percentage of viable, necrosis, late apoptotic, and early apoptotic cells was determined using quadrant statistics. The percentage of gated cells in each quadrant was plotted on a bar graph. Analysis was performed using Prism version 8 (GraphPad Software, USA) and expressed as percentage (%). Note: AV^−^/PI^−^ viable cells; AV^+^/PI^−^ early apoptosis; AV^−^/PI^+^ necrosis; AV^+^/PI^+^ late apoptosis/necrosis.

### Solubility studies

Liquid lipids containing long- and medium-chain fatty acids were screened for the solubility of CXB. The solubility of CXB was determined by adding an excess amount of the compound to 2 mL of liquid lipid in a 5-mL vial. The mixture of CXB and lipid liquid was vortexed and kept in an isothermal bath at 25 ± 0.5 °C for 24 h. After reaching equilibrium, the mixture was centrifuged at 12,045 × *g* for 15 min. The supernatant was collected and diluted with the mobile phase. The samples were analyzed in triplicate using the high-performance liquid chromatography (HPLC) method described in the “usNLCs: in vitro cellular behavior” section [36]. The solubility of CXB in solid lipids (see the “Pre-screening solubility studies: solid and liquid lipids, and surfactant” section) was determined by gradually adding an amount of solid lipid to completely dissolve 10 mg of CXB at 10 °C above the melting point of the respective solid lipid in a water bath, under magnetic stirring. The mixture was visually observed for solubilization of CXB. All measurements were performed in triplicate.

#### Solid: liquid lipid compatibility study and surfactants selection

The selected liquid and solid lipids were studied for their physical compatibility. The binary mixture in the ratio of 1:1 was filled into glass tubes. The mixture was melted at 50 °C, homogenized, and cooled to solidify at room temperature. The glass tubes were visually inspected under bright light for the absence of separate layers in the solidified lipid mass.

Surfactants are used to increase the long-term stability of usNLCs, and their selection depends on the lipid matrix because they must be compatible with the administration route and are key to the colloidal properties of the nanoparticles. Thus, the surfactant selection was based on its ability to emulsify solid–liquid binary lipid. The mixture of solid–liquid lipid (100 mg) was dissolved in 3 mL of methylene chloride and added to 10 mL of 5% surfactant solutions under magnetic stirring. The organic phase was removed at 40 °C and the resulting suspensions were diluted with tenfold Milli-Q water. Percentage transmittance of the resulting samples was observed using a UV spectrophotometer at 510 nm.

#### Risk assessment

The identification of critical material attributes (CMAs) and critical process parameters (CPPs) influencing the development of optimized NPs is a prerequisite for the quality by design (QbD) approach. Among the tools, failure mode, effects, and criticality analysis (FMECA) is a widely used approach for risk assessment. It can be used to perform a quantitative risk assessment to identify the CQAs that have the highest probability of product failure. FMECA consists of identifying potential failure modes, consequences, and causes, classifying, and ranking each failure mode according to the risk priority number (RPN = Severity × Occurrence × Detection), which provides a number between 5 and 125. An RPN above 100 is considered a high-risk factor associated with a higher failure mode. In this way, it is possible to prioritize actions and implement a control risk status for each critical activity. This tool can also establish and optimize maintenance plans for repairable systems and/or contribute to control methods and other quality assurance procedures. Benefits of FMECA risk analysis include increased reliability, better quality, improved safety, and cost savings, encompassing reduced development time and non-value-added operations. The most critical CQAs for usNLCs development obtained from FMECA were subjected to a design planning.

### Optimization of usNLCs

usNLCs were prepared using the hot-high pressure homogenization (HPH) technique, as previously described and optimized [37]. Briefly, the lipidic phase at 15% w/w consisting of various combinations of liquid and solid lipids, and 1% w/w of the oily surfactant Lipoid S 75^®^, see Tables 1 and 2, was prepared and heated to 50 °C. In parallel, the aqueous surfactant phase containing Tween^®^ 80 (T80, 5% w/V) or Kolliphor^®^ 40 (KRH40, 5% w/V) was prepared and heated up to 50 °C. The aqueous solution was added to the lipid phase, and the mixture was then homogenized using an Ultra-Turrax X 10/25 (Ystral GmBh, Dottingen, Germany) at 24,000 rpm for 1 min. The pre-emulsion formed was further processed by hot-HPH for 7.5 min at 1000 bar (Emulsiflex C-3, Avestin, Mannheim, Germany), and the resulting nanoparticles were cooled to 4 °C [28]. For the preparation of CXB-loaded usNLCs, the addition of CXB (5% w/w) was carried out in the initial molten lipid phase. usNLCs were further purified by ultrafiltration-centrifugation using centrifugal filter units (Amicon^®^ Ultra 4-, Millipore, Germany) with a 50 kDa molecular weight cut-off. Five milliliters of usNLCs were added to the centrifuge filter unit and centrifuged for two cycles of 30 min at 5000 × *g* and 4 °C. After each step, the usNLCs formulations were resuspended in ultra-purified water/PBS. The free drug in the aqueous phase collected in the outer chamber of the centrifugal filter was suitably diluted in the mobile phase, filtered through a 0.22-µm membrane, and determined by HPLC.

**Table 1:** Composition of the usNLCs formulations

**CODE**	**Lipidic compound(s)** **(15% w/w)**	**Aqueous surfactant(s) (2.5% or 5% w/w)**	**Oily surfactant (1% w/w)**
T80 S:S:C	Sup:S:C (25:25:50)	T80	LS75
KRH40 S:S:C	Sup:S:C (25:25:50)	KRH40	LS75
T80 P:S:C	P:Sup:C (25:25:50)	T80	LS75
KRH40 P:S:C	P:Sup:C (25:25:50)	KRH40	LS75

Key: Sup, Suppocire^®^ NB; S, Softisan^®^ 601; C, Capryol™ PGMC; P, Precirol^®^ ATO 5; T80, Tween^®^ 80; KRH40, Kolliphor^®^ RH40; LS75, Lipoid^®^ S75

**Table 2:** Two-level, two-variable, 2^2^, full factorial design for the optimization of the usNLCs composition (independent variables and respective codification)

**Independent variables**	**Levels**	
	** − 1**	** + 1**
**Lipid composition (LC)**	SC	SSC
**Type of surfactant (TS)**	KRH40	T80
**Responses**	Physicochemical characteristics: PS, PdI, ZP, and DL Performance in vitro*:* release studies, and cytotoxicity studies (at 24 h and 72 h)	

Key: *SS*, Suppocire^®^ NB: Softisan^®^ 601; *S*, Suppocire^®^ NB; *C*, Capryol™ PGMC; *T80*, Tween^®^ 80; *KRH40*, Kolliphor^®^ RH40; *PS*, Particle size; *PdI*, Polydispersity index; *ZP*, Zeta potential; *DL*, Drug loading

#### Experimental design

The optimization of the usNLCs, in what concerns the nanoparticle composition, was performed by a DoE methodology. A two-level, 2^* k*^ full factorial design was considered to explain the main effects and interaction of lipid composition (LC) and type of surfactant (TS) on the physicochemical characteristics (PS, PdI, ZP, and DL) and on the performance in vitro (release studies and cytotoxicity studies at 24 h and 72 h), see Table 2. The lipid composition, LC (factor 1), was inspected considering the following combinations: Suppocire^®^ NB:Softisan^®^ 601:Capryol™ PGMC and Suppocire^®^ NB:Capryol™ PGMC, represented by the coded levels + 1 and − 1, respectively. Also, the type of surfactant, TS (factor 2), was analyzed regarding Tween^®^ 80 and Kolliphor^®^ RH40, at a concentration of 5% w/V, corresponding to + 1 and − 1, respectively.

This mathematical tool allows to obtain a high amount of information requiring a relatively reduced number of experiments. Both Student *t*-test and ANOVA were performed to test whether the terms in the regression model were statistically significant and to assess the validity of the model fit, respectively. A value of *p* < 0.05 was considered statistically significant. The following regression model equation was applied:

$$y\hspace{0.17em}=\hspace{0.17em}\beta_{0}\hspace{0.17em}+\hspace{0.17em}\beta_{1}x_{1}\hspace{0.17em}+\hspace{0.17em}\beta_{2}x_{2}\hspace{0.17em}+\hspace{0.17em}\beta_{12}x_{1}x_{2}$$

where *β*_0_ is the response in the absence of effects, *β*_1_ and *β*_2_ are the coefficients of the respective independent variables, and *β*_12_ the interaction term. A total of 4 formulations were considered. The fitted models were retrieved using JMP Pro 16 Software (Cary, NC).

#### Characterization of lipid nanoparticles

##### Particle size and zeta potential analyses

The particle size (PS) and polydispersity index (PdI) were determined by dynamic light scattering (DLS). These parameters were measured using a Zetasizer Nano ZS (Malvern, Worcestershire, UK) at a 173° detection angle and a temperature of 25 °C, with the average hydrodynamic particle size (*z*-average) being determined through the cumulants method, using Zetasizer 7.02 software.

The zeta potential was determined by electrophoretic light scattering at 25 °C using the Helmholtz-Smoluchowsky approximation. The samples were diluted 100 times with ultrapurified water and analyzed three times. The results were presented as mean ± standard deviation.

##### Drug loading

The drug loading (DL) was determined indirectly by measuring the amount of free drug in the dispersion. The drug loading is the percentage of entrapped drug divided by lipid mass into a matrix and is given by

$$DL \left(\%\right)=\frac{W_\text{Total amount of drug}-W_\text{Total amount of free drug in the dispersion}}{W_\text{Total amount of lipid}}\times 100$$

The total amount of CXB was estimated using a specific volume of the usNLCs dispersion after being adequately diluted in the mobile phase and heated at 60°C for 15 min. The dispersion was then centrifuged for 5 min at 12,500 × *g* in a Minispin^®^ (Eppendorf Ibérica S.L., Madrid, Spain). The supernatant was collected, filtered through a 0.22 µm membrane, and analyzed by HPLC. The amount of encapsulated CXB was determined in each freshly prepared formulation sample. Each sample was analyzed three times using the HPLC method described in the “Instrumentation and chromatographic conditions” section.

##### Drug release studies

The dialysis bag method was used to study the CXB release behavior from usNLC formulations. For that, dialysis membranes were kept overnight in ultrapurified water to ensure the wetting of the membrane. A volume of 2 mL of the formulation was inserted into the dialysis bags and subsequently placed in 100 mL of dissolution medium at pH 7.4 (simulating pH systemic circulation). The medium was kept under magnetic stirring throughout the test period, and the temperature was set at 37 °C. The best formulation was tested at 45 °C (pH 7.4) to understand the release behavior under hyperthermia conditions. A sample volume of 750 μL of buffer medium was withdrawn at 0.5, 1, 2, 3, 6, 9, 12, 24, 30, 36, 48, 56, 60, and 72 h, and replaced by an equal volume of fresh medium. The samples were suitably diluted with mobile phase and analyzed by HPLC, as described in the “Instrumentation and chromatographic conditions” section. The dissolution profiles were obtained by plotting the cumulative percentage of drug released against time, estimated according to

$$\begin{aligned}\mathrm{Release}(\mathrm{\%})=& (\mathrm{drug\, amount\div released\, total\, amount}\,\\& \mathrm{of\, drug\, in\, formulation)} \times 100\end{aligned}$$

## usNLCs: in vitro cellular behavior

U87 cells were cultured in DMEM medium, supplemented with 10% (v/v) FBS, 1% (v/v) penicillin–streptomycin solution, and sodium bicarbonate. Cells were maintained at 37 °C in a humidified atmosphere containing CO_2_ (5%). Briefly, 20 × 10^4^ cells/well were seeded in a 96-well plate and incubated for 24 h or 72 h after replacing the medium with increasing concentrations of usNLCs. Subsequently, the medium was removed, and the cell viability was determined by resazurin assay, as described in the “Cell viability assay” section.

## Instrumentation and chromatographic conditions

The HPLC analysis of CXB was carried out using a Shimadzu LC-2010C HT apparatus (Shimadzu Co., Kyoto, Japan) equipped with a quaternary pump, a CTO-10AS oven, and an SPD-M2OA detector. A Kinetex^®^ EVO C18 column (Torrance, USA), with 5 µm particle size, 4.6 mm internal diameter, and 150 mm length, was used for the analysis in an oven at a temperature of 35 °C. Chromatographic analysis was conducted in isocratic mode. The mobile phase consisted of a mixture of aqueous solution of glacial acetic acid (2% v/v):acetonitrile in the ratio of 55:45 (v/v) and a flow rate of 1.2 mL/min [36]. A run time of 13 min was established, and CXB was eluted at 11.1 min. The detection was carried out at 250 nm, and an injection volume of 10 μL was used for all standards and samples. A stock solution (1 mg/mL) was firstly prepared, followed by the calibration standards (0.5–100 µg/mL) and quality controls (0.5, 1.5, 50, and 100 µg/mL) containing the analyte. The results were processed using a Shimadzu LC-solution version 1.12 software.

## Statistical analysis

Two-way ANOVA with Tukey’s multiple comparison test was employed using Prism version 8 (GraphPad Software, San Diego, CA, USA) to assess the statistical significance of the differences among drugs (*p* < 0.05). All experiments were performed in triplicate.

## Results and discussion

The focus of this work concerned two main objectives: addressing novelty stemming from the use of CXB as repositioning strategy and designing an enhanced formulation on the basis of a quality by design framework. Our approach for selecting CXB as repurposed drug was grounded on a tripartite perspective, described in the sections that follow:

- (i) Evidence-based on literature analysis, specifically considering our preliminary publications [38, 4], wherein an extensive literature search was conducted to list drugs that are candidates for repurposing based on their preferential damage to GB cells by various mechanisms. CXB has already been tested as anticancer drug in clinical trials and showed positive results, which motivated its selection for potential use in GB treatment [4].
- (ii) Evidence-based on bioinformatics tools, specifically resorting to open access databases, aiming to enlarge the knowledge on the biological pathways and molecular processes inherent to cell death mechanisms, along with predicting CXB targets with the genes/proteins connected with GB.
- (iii) Evidence-based on experimental cellular viability studies, resorting to a set of four glioma cell lines to infer the cytotoxicity of CXB and the respective comparison or potential combination with TMZ, as standard of care, and identify the one that could provide the best sensitivity and discriminatory power toward the formulation development and subsequent in vivo performance assessment.

### Predicting CXB targets with the genes/proteins connected with glioblastoma

Although TMZ is the first-line chemotherapy for GB patients, at least 50% of patients do not respond to this drug [39]. This failure is due to mechanisms of resistance acquired by GB cells, among others related to the enzyme O^6^-methylguanine DNA methyltransferase (MGMT), which removes TMZ-induced O^6^ methyl adducts to allow DNA replication to continue [39–41]. Such outcome has dictated the investigation of therapeutic alternatives for GB treatment, considering a repurposing drug strategy. Drug repositioning can be a complex process that requires several steps, involving different types of data analysis and experimental validation. The latter is associated with high costs and the success rate is usually minimal. For this reason, the use of bioinformatics tools has greatly improved the prospects for developing hypotheses and models for drug mechanisms of action, assisting in structure-guided drug target prediction and repositioning [29, 32, 42–45]. Bioinformatics provide insights into therapeutic options at the drug target and disease levels, which facilitates the understanding of drug pathways yet to be exploited. This has created the opportunity to investigate the potential of drug reuse prior to experimental testing, which is particularly attractive for cancer research. Thus, it was possible to study a range of genes and variants involved in GB and to map the full spectrum of potential interactions between the compounds (CXB and TMZ) and their targets.

**Fig. 1:**
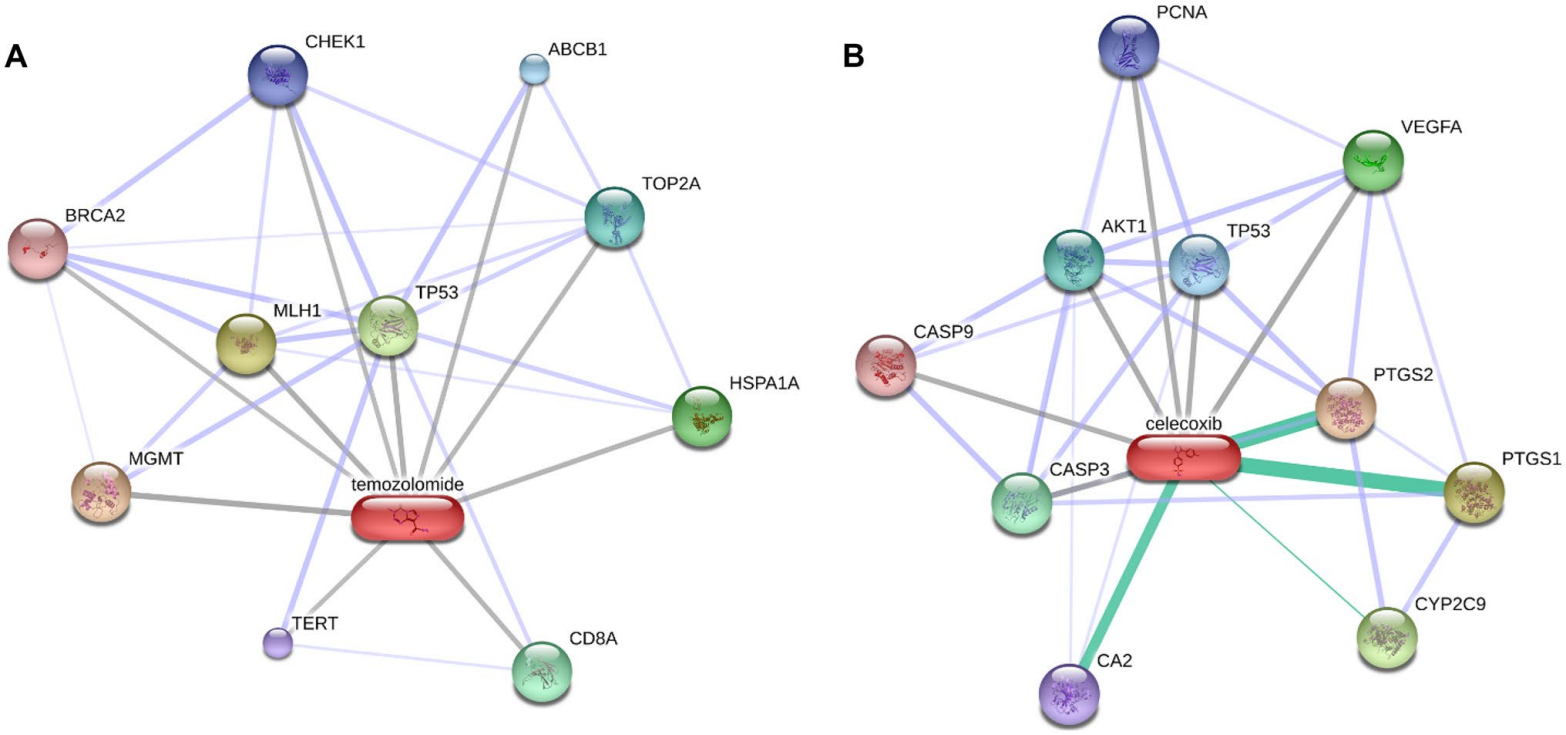
Protein–protein network between proteins encoded by the genes targeted by TMZ (**A**) and CXB (**B**). Proteins are identified with the respective gene name

In the present work, the main purpose of resorting to bioinformatics was to compare the mechanism of action of the standard of care (TMZ) used in GB treatment with that of a repositioned drug (CXB), and predict potential synergistic effects, or even understand whether there are common targets. To this end, DisGeNET was used to characterize the GB gene expression signature, while STITCH was used to characterize the CXB/TMZ-protein interaction network and explore the relevant biological processes. The protein–protein network and biological processes of the 10 TMZ and 10 CXB drug protein targets (DPTs) were generated by STITCH (Fig. 1 and Table 3). Among the biological processes identified for TMZ, the regulation of DNA metabolic process (*p* = 3.08 × 10^−5^) was considered the most relevant, with six DPTs associated (*BRCA2*, *CHEK1*, *MGMT*, *MLH1*, *TOP2A*, *TP53*). As for CXB, the most important process was glial cell apoptosis (*p* = 4.46 × 10^−5^), and there were three DPTs identified (*CASP3*, *CASP9*, *TP53*). The latter points out to a possible linkage of the CXB action on GB.

**Table 3:** List of biological processes associated to TMZ and CXB

**Temozolomide**		
**Pathway description**	**False discovery rate**	**Matching proteins in the network (labels)**
Regulation of DNA metabolic process	3.08 × 10^−5^	*BRCA2, CHEK1, MGMT, MLH1, TOP2A, TP53*
Replicative senescence	0.000131	*CHEK1, TERT,* *TP53*
Cell aging	0.000147	*BRCA2, CHEK1, TERT, TP53*
Meiotic metaphase I plate congression	0.000725	*BRCA2,* *MLH1*
Chromosome organization	0.00341	*BRCA2, CHEK1, MLH1, TERT, TOP2A, TP53*
**Celecoxib**		
**Pathway description**	**False discovery rate**	**Matching proteins in the network (labels)**
Glial cell apoptotic process	4.46 × 10^−5^	*CASP3, CASP9,* *TP53*
Response to lipid	9.5 × 10^−5^	*AKT1,* *CA2, CASP3, CASP9, PCNA,* *PTGS1, PTGS2*
Response to radiation	0.000126	*AKT1,* *CASP3, CASP9, PCNA, PTGS2, TP53*
Cellular response to abiotic stimulus	0.000314	*AKT1,* *CASP9*, *PCNA, PTGS2, TP53*

**Fig. 2:**
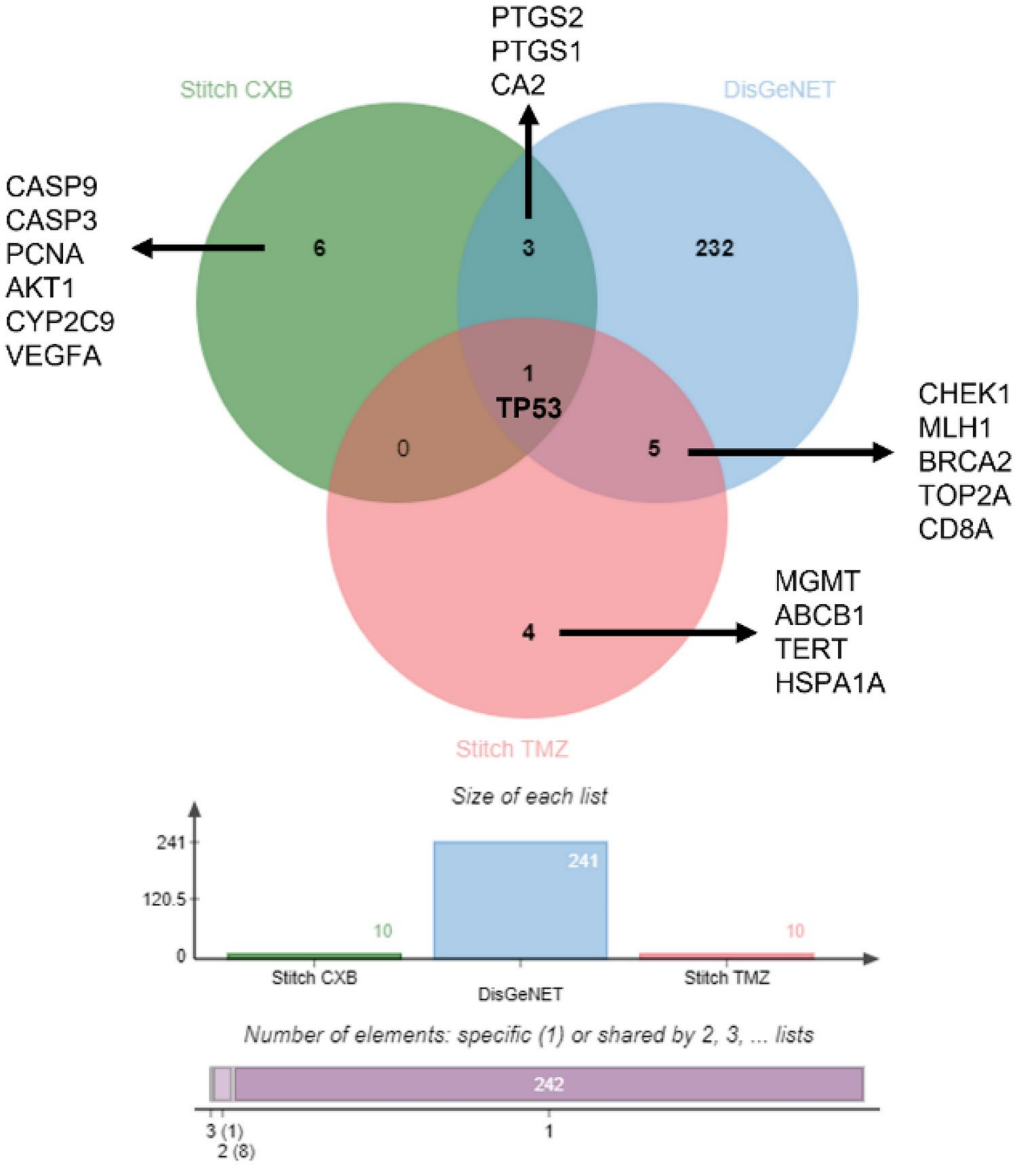
Venn diagram of genes evaluated in the set of GB, CXB, and TMZ responsible for protein expression. Of the 261 genes described in the literature, 241 were associated with GB, 10 appear to be specific for CXB, and 10 for TMZ. Comparative analysis was performed using jVenn software

The overlapping genes between CXB, TMZ, and GB in human samples were identified by a combination of STICH/DisGeNET and constructed using jVenn software. Venn diagram was used to retrieve the common proteins (Fig. 2). The intersection of datasets was performed to find out important protein targets of CXB as a potential drug in GB treatment. Evaluation of CXB targets with all reached proteins/genes related to GB (all glioblastoma-related genes ∩ CXB targets) was carried out. Evaluation of TMZ targets with major proteins/genes was also performed using the STITCH tool (all glioblastoma association genes ∩ TMZ targets). Venn diagrams showed that only three proteins (*CA2, PTGS1*, and* PTGS2*) were found to be common (CXB ∩ glioblastoma), whereas only one protein (TP53) was found to be common in CXB ∩ glioblastoma ∩ TMZ. TP53 is interlinked with one of the most frequently deregulated genes in cancer, including GB, where the pathway is deregulated in 84% of GB patients [46].

**Fig. 3:**
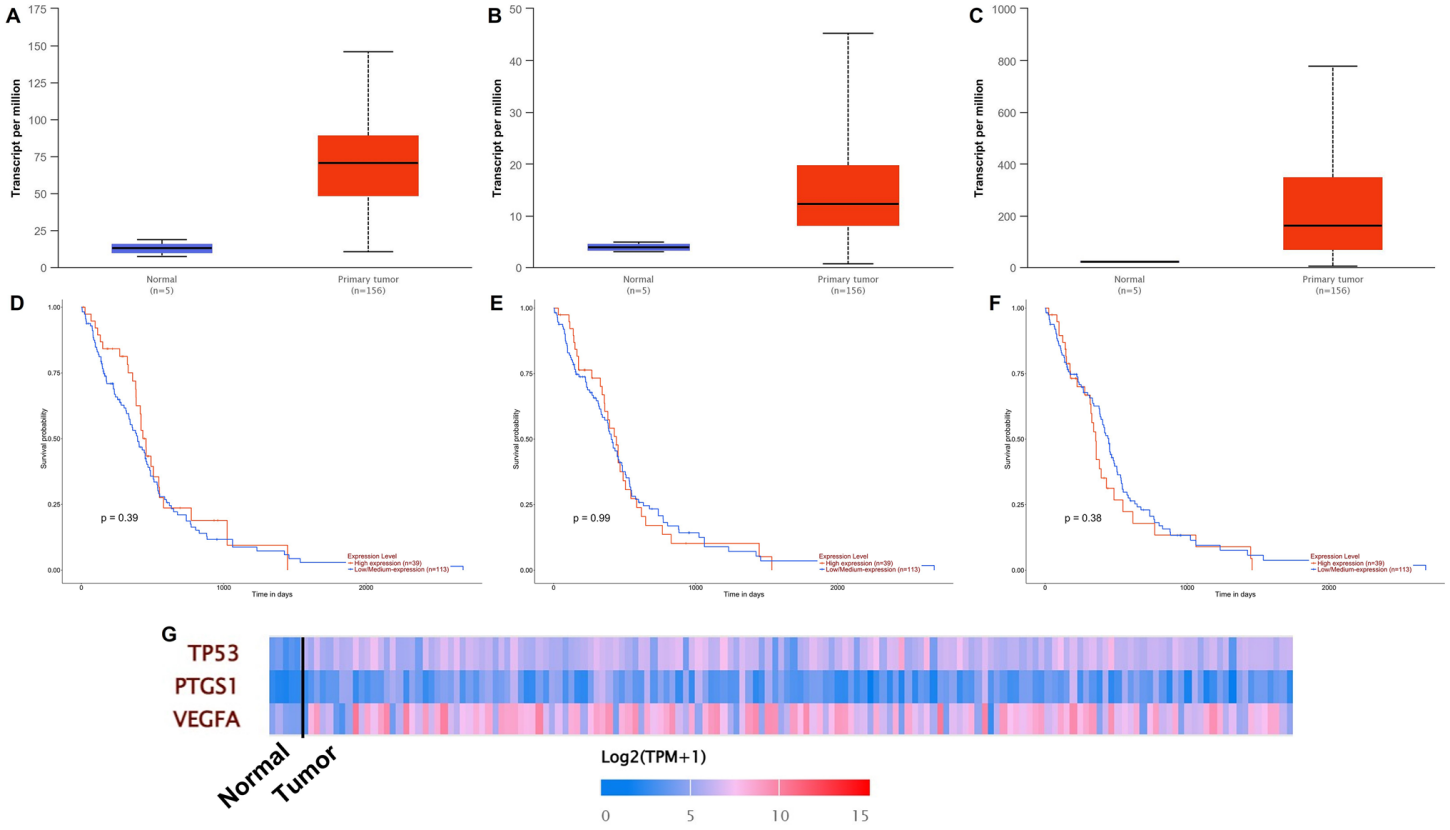
Expression prognostic value of selected proteins, targeted by TMZ and CXB (*TP53*, **A**; *PTGS1*, **C**; *VEGFA*, **E**), in GB patients. Correlation analysis of *TP53* (**B**), *PTGS1* (**D**), and *VEGFA* (**F**) expressions with the overall survival of GB patients in TCGA–GB. Heatmap with the three genes mutational frequencies in GB (**G**)

Based on the previous results (jVenn diagram and protein–protein network), three genes (*TP53*, *PTGS1*, and *VEGFA*) were selected to explore their expression status in GB compared with normal tissue using the UALCAN databases. The validated results of the expression levels, prognostic values of the upregulated genes in GB, and respective heatmap are shown in Fig. 3. Highly significant gene expression levels of *TP53, PTGS1*, and *VEGFA* in GB tissue can be observed (*p*-value of < 0.001, Fig. 3A, C, and E). To shed light on the relationship between the overexpression of the three genes and the overall survival of GB patients, Kaplan–Meier (KM) survival analysis was performed at TCGA GB (Fig. 3B, D, and F). Only *PTGS1* was significantly correlated with the worst survival of glioma patients (*p* = 0.99). This suggests that higher expression levels of *PTGS1* at diagnosis can be considered an unfavorable prognostic gene that may shorten the overall survival of GB patients. Although KM survival analysis showed that *TP53* and *VEGFA* were not associated with significant prognosis in GB, patients with high expression had lower survival rate than those with low expression (*p*-value < 0.05). The mutation frequencies of the three genes in normal and GB tissues are shown in the heatmap (Fig. 3 G). Only *VEGFA* exhibited a significantly higher mutation frequency in GB. Despite this pattern, a large impact of both *TP53* and *VEGFA* on the transcriptome is described. Understanding the relationship between disease and drug-associated gene set/biological processes was deemed of utmost importance to determine a more robust druggable activity for CXB in GB.

### Repurposing CXB for glioblastoma

Several studies have shown the benefit of COX-2 inhibition in numerous cancers, including GB. Thus, a selective COX-2 inhibitor, CXB, was investigated to potentially overcome the resistance mechanisms and improve patients’ quality of life.

**Fig. 4:**
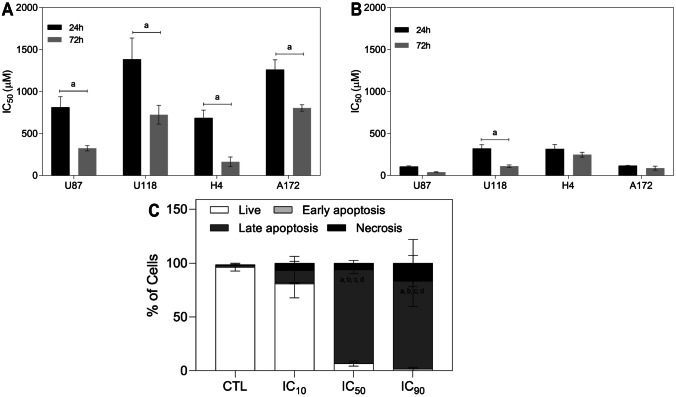
In vitro cytotoxic effect, as IC_50_, of TMZ (**A**) and CXB (**B**) on different glioma cells, at 24 h and 72 h (^a^* p* < 0.0001). Data are expressed as mean ± standard deviation, SD (*n* = 3). (**C**) Effects on cell death profile of U87 cells with different CXB concentrations (IC_10_, IC_50_, and IC_90_). After 4 h, cells were stained with annexin V (AV) and propidium iodide (PI), and the percentage of cell death was evaluated. These data are representative of three independent experiments. Live: ^a^ *p* < 0.0001 vs. at CTL (control); ^b^ *p* < 0.0001 vs. at IC_10_; late apoptosis: ^c^ *p* < 0.0001 vs. at CTL; ^d^ *p* < 0.0001 vs. at IC_10_

In the current study, the resazurin assay was used to evaluate the cell growth inhibition activity of the TMZ and CXB against four human glioma (U87, H4, A172, and U118) cell lines for 24 and 72 h. The IC_50_ values are presented in Fig. 4A, B. As expected, both CXB and TMZ significantly inhibit the growth of glioma cells in a dose- and time-dependent manner. Reported literature IC_50_ values for TMZ in glioma cells show wide variations [40, 47, 48], while for CXB, literature values are similar to those corresponding to our results [9, 49, 50]. For both drugs, IC_50_ also depends on cell type. CXB exhibits high sensibility for U87, followed by U87 > A172 > H4 > U118, while for TMZ the order is U87 ~ H4 > A172 > U118. H4, a non-tumorigenic cell line, showed high sensitivity to TMZ. CXB exhibited lower IC_50_ values (below 400 μM) than TMZ for all glioma cells. The results indicated a higher sensitivity of U87 for both drugs. For this reason, in the subsequent studies, only U87 cells were used to better discriminate the cytotoxic effects. The next step concerns the study of the potential synergistic effect between CXB and TMZ.

#### CXB and TMZ: is there a synergistic effect?

The combination of two or more therapeutic agents targeting multiple pathways simultaneously is gaining interest and showing promising results in cancer therapy. The presence of several independent or compensatory mechanisms at the cellular level makes synergistic drug-drug interaction attractive but highly challenging.

U87 cells were treated with various TMZ concentrations plus CXB, and cell viability was calculated. Chou-Talalay calculation was then performed, and the results for CI (see Eq. (3.1.1)) were 3.52 and 2.06, respectively, at 24 and 72 h. Although the results showed cell death when the drugs were used together, the CI values were higher than 1, indicating that there is no incremental effect between drugs. The literature corroborates this result and shows other in vitro studies reporting the same effect [51]. Thus, only CXB was used for the subsequent studies.

#### Cell death mechanisms

The cell viability studies showed that the treatment with CXB induced a significantly higher cell death than TMZ, suggesting that CXB could be an excellent candidate to eliminate glioma cells effectively. However, the cell viability results do not provide information about the cell death mechanism after cell treatment. Therefore, the cell death mechanism was investigated using the annexin V/propidium iodide (PI) assay by flow cytometry. Annexin V and PI detect cell death by different indicators, namely, binding to phosphatidylserine and DNA, respectively. Phosphatidylserine is located in the outer leaflet layer of the cell membrane. It is translocated to the outer layer only when caspases involved in apoptotic events cleave the membrane. Thus, viable cells cannot bind to annexin V, but when cells are in the early apoptosis stage can attach to it. In viable cells or cells in the early stage of apoptosis, the plasma membrane is intact, preventing the passage of PI through the membrane. PI stains the nucleus of non-viable cells that are in a later stage of apoptosis, indicating the presence of necrosis and late apoptosis.

To investigate the cell death mechanisms, U87 cells were treated with three concentrations (IC_90_ = 22 µM; IC_50_ = 108 µM; IC_10_ = 195 µM) of CXB for 4 h. After this period, annexin V/PI were applied. Figure 4C shows the results obtained by flow cytometry. The percentage of live cells in control and IC_10_ was higher and statistically different than IC_50_ and IC_90_ (*p* < 0.0001). Cells treated with higher drug concentrations (IC_50_ and IC_90_) promoted a higher percentage of cell death, represented by late apoptosis, than control and IC_10_. The early apoptosis is not weighting in all the concentrations, while late apoptosis is the principal mechanism of cell death after 4 h of CXB treatment. The apoptotic fraction was directly related to the selected CXB concentration. As the CXB dose increased, the proportion of apoptotic cells also increased (*p* < 0.0001).

There is a significant decrease in cell viability for the three concentrations compared with the results obtained for untreated cells. These findings suggest that the treatment with CXB can reduce cell viability through the activation of pathways involved in the regulation of apoptotic events. The flow cytometry results are consistent with the cytotoxicity determined by the resazurin assay. The in vitro effects of CXB in U87 cells induce apoptosis and cell proliferation decrease.

### Designing a nanoformulation template

Delving into nanoformulation template, a quality target product profile (QTPP) was first constructed to summarize the quality characteristics of usNLCs for maximum therapeutic efficacy (Table 4) [52–54]. Afterward, the critical quality attributes (CQAs) were thoroughly identified bearing in mind their impact on the desired in vitro and in vivo performance of usNLCs. Controlling critical attributes is essential to the quality product and keeping variability within acceptable limits. Table 4 lists the potential CQAs and the respective justifications. The selection of CQAs was carried out as per prior literature, knowledge, and by establishing a FMECA (Table 5). The latter identifies the estimated risk associated with a specific cause and prioritizes the action to be taken to reduce the risk. This is carried out by the calculation of the risk priority number (RPN) obtained from the product of severity, probability of occurrence, and detectability. This ranking is essential to identify the more relevant factors to be involved in the screening design. An RPN above 100 was considered for identifying a high-risk factor.

**Table 4:** Quality target product profile (QTPP) and critical quality attribute (CQAs) elements of the CXB-usNLCs

**QTPP element**	**Target**		**Justification**
Indication	Glioblastoma		GB is one of the most aggressive central nervous system tumors due to its invasive nature and genetic and epigenetic variability, evidencing resistance to currently used forms of therapy.
Formulation type	Ultra-small nanostructured lipid carriers (usNLCs)		usNLCs as a drug carrier system has advantages over other colloidal delivery systems, including biocompatibility, biodegradability, protection of incorporated active ingredients against chemical degradation, high drug loading capacity without drug expulsion during storage, ability to co-encapsulate two or more anticancer agents, and controlled release assigned to the solid matrix enable to achieve and maintain therapeutic concentrations over a more extended period of time at the target site. usNLCs are also beneficial at a systemic level, exhibiting therapeutic advantages, such as longer circulation half-life, improved pharmacokinetics, and reduced side effects. Their lipid composition and small size ensure close contact with lipid bilayers, improving drug delivery across biological barriers. The surface of usNLCs can be modified, thus enhancing target specificity. A high lipid content based on a low melting point lipid matrix is envisioned to assign thermosensitive feature to the formulation.
Pharmacokinetics	Improved absorption, bioavailability, and pharmacokinetic parameters compared to the free drug and/or encapsulated drug		Necessary to achieve the desired therapeutic effect.
Administration route	Parenteral administration, preferentially intravenous administration (IV)		Intravenous administration allows the highest bioavailability of therapeutic agents, associated with rapid onset, without the first-pass metabolism and avoiding the aggressive gastrointestinal environment.
**Quality attribute**	**Target**	**CQAs?**	**Justification**
Toxicity	GB cells	YES	Specific to GB cells, decreasing the side effects of the chemotherapeutic treatments.
Raw materials	Non-toxic, biocompatible	YES	Excipients used in formulations for parenteral administration should display appropriate biocompatibility and biodegradability, thus ensuring the safety of the pharmaceutical product.
Particle size	< 100 nm	YES	A critical parameter that significantly affects the permeation across the BBB and blood–brain tumor barrier (BTB). Fenestrations in BBB endows it with substantially higher permeability. Depending on the GB stage, regions in the brain present fenestrations whose size is variable and can increase to one micron. Thus, the BTB is susceptible to nanocarriers accumulation through the enhanced permeability and retention (EPR) effect, with a preferential concentration in the tumor tissues.
Zeta potential	Compatible with IV administration	YES	Zeta potential (ZP) is related to particle stability in a determined medium, and affects the stability of the formulation, preventing nanoparticle aggregation. ZP value higher than |30| mV is generally considered appropriate to evaluate a dispersion as stable. However, for intravenous administration, the more appropriate value of ZP is neutral to avoid protein binding on the usNLCs surface.
Active targeting	Ligands specific to targeted tissues, e.g., cell-penetrating peptides and tumor-targeting peptides	YES	Peptides are described as molecules with a high affinity to HBMEC or U87 cells, improving the delivery of drugs specifically to the brain and minimizing drug delivery at non-targeted tissues or with fewer off-target effects. Despite not addressed in this former study, the targeting approach involves the attachment of ligands to the carrier’s surface by electrostatic interaction or covalently.
Stability	Long-term physical, chemical, and biological stability	YES	Physicochemical stability of usNLCs during the storage period is a quality requirement to ensure therapeutic performance of the usNLCs, being also a crucial requirement for marketing authorization. Changes in the formulations, such as protein corona formation, can affect the drug release and the therapeutic effect of the formulation.
Load	Celecoxib	YES	Repurposing drug approach: CXB was the first drug belonging to the class of selective cyclooxygenase-2 (COX-2) inhibitors to be approved by the Food and Drug Administration (FDA) in 1998. CXB, an anti-inflammatory and analgesic drug, is used to block the synthesis of several pro-inflammatory prostanoids, including prostaglandins (PGs) and thromboxanes (TXs). These are the end products of fatty acid metabolism produced by COX-enzymatic activity. These mediators are critical for pathological and physiological processes, including cancer. CXB has been studied in the oncological field due to its potential anticancer properties. Overexpression of COX-2 is found chronically at various stages of carcinogenesis, resulting in higher PG levels in neoplastic tissue. The presence of inflammatory cells is a stimulus for tumorigenesis that increases COX-2 enzyme expression, leading to the activation of various mechanisms involved in cancer progression [4].
Dosage strength	Maximum	YES	This strength is within the minimum effective concentration of CXB required for antitumoral effect as per literature and preclinical investigations.
Dissolution profile	Sustained release	YES	Reduced release of CXB at pH 7.4 < 25% (plasma), and release of a larger quantity of CXB at the tumor site. The sustained release allows effective blood concentration of the drug over a longer period of time than conventional formulations.
Cell viability	IC_50_ HBMEC > IC_50_ U87	YES	Improved safety when compared to glioma cells, increasing the specificity of the nanoparticles.
Cellular uptake	Maximize	YES	The internalization of nanoparticles in both cells (HBMEC and U87 cells) is crucial for their therapeutic effect.

**Table 5:**
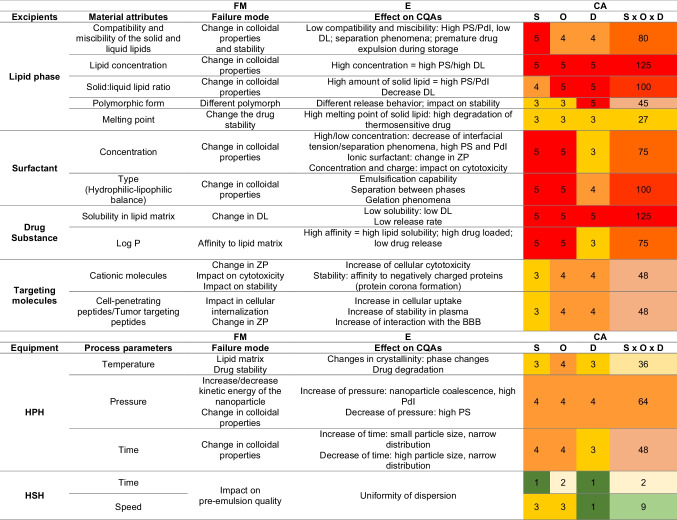
FMECA illustrating the RPN for various formulation and process variables affecting the CQAs [55, 56]. Each parameter uses a numerical scale from 1 to 5. Severity (S) = 1 (low)–5 (high); probability of occurrence (O) = 1 (low)–5 (high); detection (D) = 1 (easy)–5 (hard). The risk priority number (RNP = S × O × D) = 1–29 (low risk), 30–59 (medium risk), and 60–125 (high risk)

*CQAs* critical quality attributes, *PS* particle size, *PdI* polydispersity index, *ZP* zeta potential, *DL* drug loading, *HPH* high-pressure homogenization, *HSH* high-shear homogenization, *RPN* risk priority number

This analysis yielded the selection of toxicity, stability, particle size, zeta potential, and dosage strength as potential CQAs. Consequently, the CMAs and the CPPs, which are the most impacting variables on CQAs, were identified (Table 4). The CMAs identified for the present study were the solid and liquid lipid contents, aqueous surfactant concentration and type, drug solubility, and targeting molecules. The CPPs, such as HPH time and pressure, were previously evaluated in [28, 52]. Accordingly, an enhanced understanding of the hot-HPH method in the physicochemical properties of the usNLCs was retrieved, resorting to an examination of HPH-time and pressure as CPPs, and lipid concentration as CMA. Such findings indicated that the impact of CMAs prevailed over CPPs. For this reason, HPH-time and pressure were kept constant (7.5 min and 1000 bar, respectively). This yielded usNLCs with a high lipid content (15%w/w), reduced size (< 100 nm), and narrow size distribution (polydispersity index < 0.2).

Pre-formulation studies must be done to support an appropriate excipient selection. Based on the results of the FMECA, the solubility of CXB in solid and liquid lipids, the solid:liquid lipid ratio, and the surfactant concentration and type were selected as the most critical parameters influencing the performance of the usNLCs and were further investigated in the design of experiments (DoE). In this work, an appropriate selection of solid and liquid lipids is envisioned, focusing on the physicochemical properties of CXB, and for that reason, pre-screening solubility studies were conducted. Note that the solubility profile of CXB in a lipid matrix plays a key role in the drug loading (DL) of usNLCs. After the selection of lipids, different ratios between lipids and the surfactant were inspected in terms of colloidal properties and stability. The optimized usNLCs dispersions were assessed in terms of drug release and cytotoxicity, as biological performance outcome.

#### Pre-screening solubility studies: solid and liquid lipids, and surfactant

Solid and liquid lipids for the preparation of usNLCs were selected depending on drug solubility. A high solubility of the solid and liquid lipid will enable higher entrapment efficiency, essential for nanosystem performance. Capryol^®^ 90, Capryol™ PGMC, Capmul MCM C8, Labrafil, and Lauroglicol 90 showed suitable solubility of CXB (Table 6). Despite the high solubility of CXB in Capryol^®^ 90, this liquid lipid led to the gelation process after the usNLCs production. Therefore, Capryol^®^ PGMC was selected as the liquid lipid due to the high CXB solubility, which will contribute to the maximization of the drug encapsulation. In what concerns the solid lipid, CXB presents a low affinity to Witepsol E76, E85, and cetyl palmitate (5.5 ± 0.4 mg/g, 5.0 ± 0.3 mg/g, and 8 ± 1 mg/g, respectively); and showed high affinity to Suppocire^®^ NB (14 ± 2 mg/g), Precirol^®^ ATO (21 ± 1 mg/g), Imwitor^®^900 F (27 ± 9 mg/g), Apifil (38 ± 8 mg/g), and Softisan^®^ 601 (77 ± 13 mg/g, see Table 6). Note that all these solid lipids differ in composition and melting point.

**Table 6:** CXB solubility in liquid lipids and solid lipids (mean ± SD, *n* = 3, * *p* < 0.01; ** *p* < 0.001; *** *p* < 0.0001)

**Liquid lipid**	**CXB solubility (mg/mL)**	**Solid lipid**	**CXB solubility (mg/g)**
**Capryol™ 90**	107 ± 2*		
**Capryol™ PGMC**	89 ± 5	**Softisan**^**®**^ **601**	77 ± 13
**Capmul MCM**	81 ± 10	**Apifil**	38 ± 8**
**Labrafil**^**®**^	68 ± 14*	**Imwitor**^**®**^ **900**	27 ± 9***
**Lauroglicol**^**®**^ **90**	47 ± 3***	**Precirol**^**®**^ **ATO**	21 ± 1***
**Labrafac**^**®**^ **PG**	15 ± 9***	**Suppocire**^**®**^ **NB**	14 ± 2***
**Miglyol**^**®**^ **812**	14 ± 1***	**Cetyl palmitate**	8 ± 1***
**Labrafac**^**®**^ **WL 1949**	12 ± 2***	**Witepsol**^**®**^ **E76**	5.5 ± 0.4***
**Oleic acid**	2.3 ± 0.3***	**Witepsol**^**®**^ **E85**	5.0 ± 0.3***
**Squalene**	0.03 ± 0.01***		

Emulsifiers were used for stabilization of the lipid dispersions by reducing the interfacial tension between the lipid phase and the aqueous phase during the production of the particles. The selection of surfactants mainly depends on their compatibility with the lipid matrix, as it contributes to the control of particle size, crystallization behavior, and stability of the dispersions. For surfactant screening, emulsification capacity according to the solid–liquid lipid binary mixture (high transmittance, high emulsification capacity) and targeting properties (mainly to BBB receptors) were considered. Solutol^®^ 15, Kolliphor^®^ 188, Myrj^®^ 52, Kolliphor^®^ RH40, and Tween^®^ 80 were evaluated. All the surfactants selected demonstrate inhibition in vitro of P-gp, a receptor significantly expressed in the BBB; and Kolliphor^®^ RH40 and Tween^®^ 80 also demonstrate inhibition in vitro of MRP2, a transporter mainly expressed in the liver, kidney, and intestine, which can be relevant in the metabolization and excretion of nanoparticles [57, 58]. Besides the reported targeting ability, Tween^®^ 80 and Kolliphor^®^ RH 40 also showed the highest transmittance (73.3% and 67.6%, respectively) and were used for the subsequent studies. In addition to an aqueous surfactant, an oily phase surfactant was included. Previous results showed the best performance of Lipoid^®^ S75 instead of SPC-3 and E100 [52, 59]. So, Lipoid^®^ S75 was used as oily surfactant in the present work.

#### Lipid compatibility and ratio selection

In the selection of the solid lipid, consideration of the solubility of the drug is important, but not enough to ensure the colloidal stability and drug release. For these reasons, the effects of the five best solid lipids, in terms of CXB solubilization, on the quality of the lipid matrix were evaluated considering the impact of the surfactant. To assist the selection of a favorable nanoparticle composition, the colloidal properties were evaluated, including particle size, PdI, and zeta potential, reinforcing the importance of attaining a particle size below 100 nm, a narrow distribution, and a zeta potential in excess of |30| mV.

**Fig. 5:**
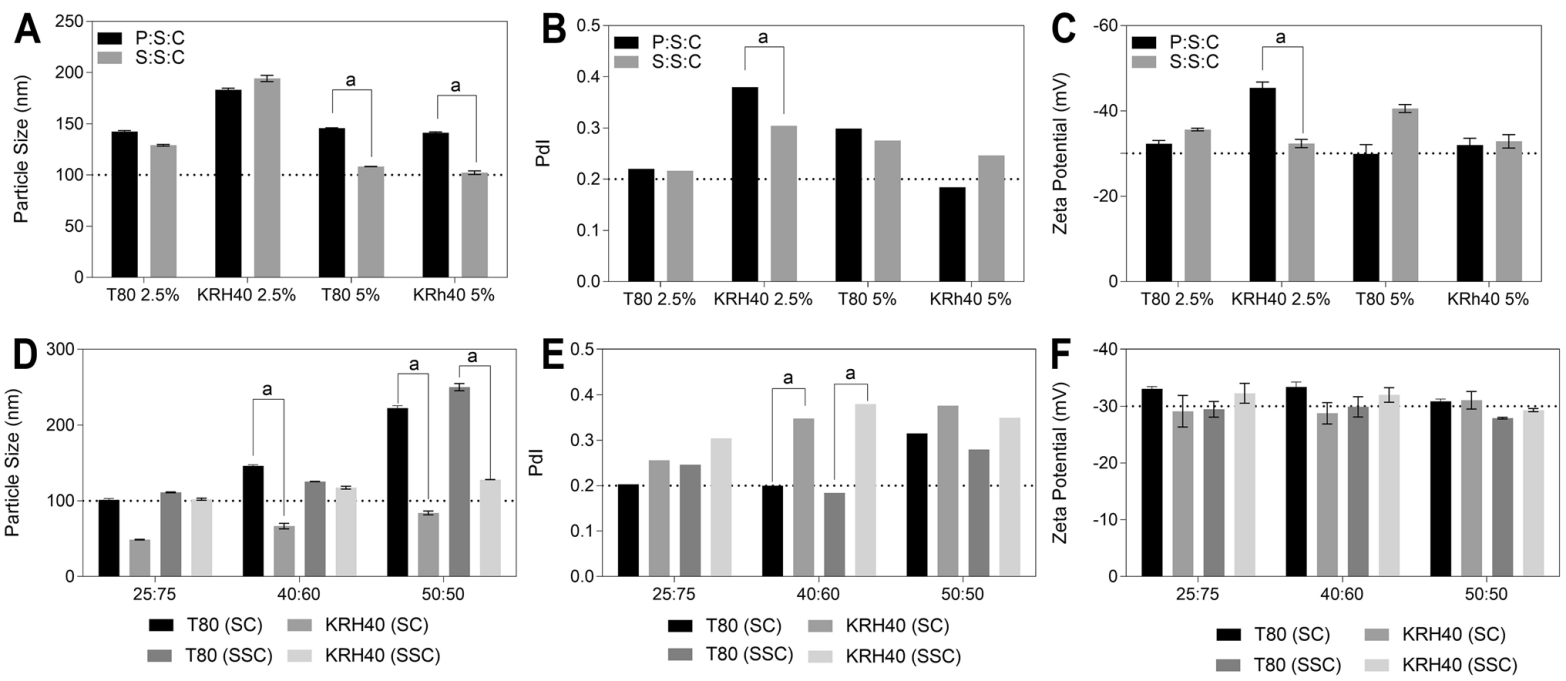
**A**–**C** Particle size, polydispersity index, and zeta potential of usNLCs considering different lipid composition (P:S:C and S:S:C) and different surfactants, Tween^®^ 80, and Kolliphor^®^ RH40 (mean±SD, *n* = 3, ^a^ *p* < 0.01). Key: P:S:C, Precirol^®^ ATO 5:Softisan^®^ 601:Capryol™ PGMC; S:S:C, Softisan^®^ 601:Suppocire^®^ NB:Capryol™ PGMC; T80, Tween^®^ 80; KRH40, Kolliphor^®^ RH40. **D**–**F** Particle size, polydispersity index, and zeta potential of usNLCs considering S:S:C and S:C as lipid composition combined with different surfactants Tween^®^ 80 and Kolliphor^®^ RH40 at different solid:liquid lipid ratios (mean±SD, n = 3, ^a^ *p* < 0.01). Note that when two solid lipids are considered, the concentration of each is half the overall value. Key: S:C, Suppocire^®^ NB:Capryol™ PGMC

Apifil and Imwitor^®^ 900 rendered a high particle size (> 150 nm). Both solid lipids were discarded and did not proceed to the next step. Softisan^®^ 601, a monoglyceride, was found to have the highest affinity for the drug, with a good compatibility with the selected oily phase (Capryol™ PGMC). Precirol^®^ ATO 5, glycerol distearate, showed good drug and liquid lipid compatibility. Suppocire^®^ NB, a hard fat, displayed a positive compatibility with Capryol™ PGMC, despite the low solubility of CXB, compared to Precirol^®^ ATO 5 or Softisan^®^ 601. However, Suppocire^®^ NB has a low melting point, 38 °C, which corresponds to the optimal phase transition temperature for thermally triggered release under hyperthermia conditions. Based on the advantages of Softisan^®^ 601, Precirol^®^ ATO 5, and Suppocire^®^ NB, eight different formulations with two different lipid compositions (S:S:C: Softisan^®^ 601: Suppocire^®^ NB: Capryol™ PGMC; P:S:C: Precirol^®^ ATO 5: Softisan^®^ 601: Capryol™ PGMC) were combined with two surfactants (Tween^®^80 vs. Kolliphor^®^ RH40, considering a concentration of 2.5% w/V and 5% w/V), see Fig. 5. The surfactant type and concentration influenced the particle size and PdI, and ZP remained above |30| mV irrespective of the composition (Fig. 5A–C). A low surfactant concentration (2.5% w/V) yielded particle sizes above 140 nm. In turn, a higher surfactant concentration (5% w/V) resulted in a smaller particle size and narrow distribution. Looking at the lipid composition (P:S:C vs. S:S:C), there were no significant differences. However, the lipid matrix considering S:S:C showed a smaller particle size (~ 100 nm) for both surfactants (see Fig. 5). For this reason, the lipid composition (lipid ratio) of Softisan^®^ 601: Suppocire^®^ NB: Capryol™ PGMC was chosen for the next studies.

The miscibility between Softisan^®^ 601:Suppocire^®^ NB, as solids, and the Capryol™ PGMC, as liquid lipid, was initially evaluated by visual inspection upon heating in different ratios (w/w): 50:50, 40:60, and 25:75, corresponding to the ratios most commonly used in other work [52]. Three different ratios were assessed with two different surfactants: Kolliphor^®^ RH40, and Tween^®^ 80 (see Fig. 5 D–F). Kolliphor^®^ RH40 conferred a smaller particle size (< 100 nm), while Tween^®^ 80 prompted a particle size larger than 100 nm, mainly with the higher amount of solid lipids and when S:C is used. Thus, a higher amount of liquid lipid was considered to improve the encapsulation of CXB, resulting in a reduction of particle size and a narrow distribution in both cases. Again, the use of different surfactants showed no differences in ZP.

In summary, Capryol™ PGMC, Softisan^®^ 601, and Suppocire^®^ NB were chosen as components of the lipid matrix, and KRH40 and T80 as surfactants. The concentration of surfactants was fixed at 5% w/w. The ideal ratio between solid and liquid lipids was 25:75, following the earlier argument.

Thus, for the subsequent studies, four different formulations and the corresponding loadings, were evaluated (Fig. 5): Suppocire^®^ NB: Softisan^®^ 601:Capryol™ PGMC + Tween^®^ 80 (SSC T80), Suppocire^®^ NB:Capryol™ PGMC + Tween^®^ 80 (SC T80), Suppocire^®^ NB Softisan^®^ 601:Capryol™ PGMC + Kolliphor RH 40 (SSC KRH40), and Suppocire^®^ NB:Capryol™ PGMC + KRH40 (SC KRH40).

#### Optimization of usNLCs by a design of experiments

The previous data clearly showed that usNLCs are highly influenced by the lipid matrix composition and surfactant, considering both type and concentration [60, 61]. During the lipid screening, it was concluded that the optimal components for usNLCs formulation are Softisan^®^ 601:Suppocire^®^ NB:Capryol™ PGMC with a 12.5:12.5:75 solid lipid:liquid lipid ratio. To evaluate the influence of each solid lipid composition and to find the best conditions for the optimal formulation, a full factorial experimental design was used. With the application of a two-level, two-variable, 2^2^ full factorial planning, over the optimal lipid composition (SC or SSC), surfactant type (KRH40 or T80), as CMAs, information about the interactions of factors can be provided. Since the concentration of lipid composition and the surfactant concentration were previously fixed, they were not considered CMAs. Note that the identification of the best formulations aimed at (i) improving the drug solubility, therefore prompting a higher entrapment efficiency; (ii) producing a lipid matrix with a low melting point to ensure a higher local release of the drug (hyperthermia approach, resulting in a triggered release in tumor cells); and (iii) enhancing the cytotoxicity effect in U87 cells. Table 7 shows the quality parameters selected as CQAs and the performance in vitro. These must be examined together with Fig. 5 D–F, where the effect of different surfactants and lipid matrix composition in CXB loaded-usNLCs is plotted. usNLCs with T80 as surfactant exhibited larger particle size and PdI, regardless of lipid composition (as seen previously). In contrast, KRH40 rendered a smaller particle size and PdI. For both surfactants, the ZP was close to − 30 mV, pointing out to formulation stability. According to the results, CXB addition did not interfere with the colloidal parameters, such as PS, PdI, and ZP. Several drug loadings, 2.5, 5, 7.5, and 10% w/w, were tested (data not shown). However, only usNLCs with 2.5% and 5% w/w were considered to have stability without drug expulsion in storage. Thus, 5% w/w of CXB was selected, which indicates that approximately 7.5 mg/mL of the drug was present in the formulation. DL of the CXB loaded-usNLCs was higher to SC combination than SSC (Table 7). Capryol™ PGMC contributed to better drug encapsulation, having reduced the particle crystallinity, and imparted better stability.

**Table 7:** Two-level, two-variable, 2^2^, factorial design for the optimization of the usNLCs formulations and representation of quality parameters selected as CQAs, including formulation physicochemical characteristics (PS, PdI, ZP, and DL; *n* = 3 ± SD, standard deviation) and the performance in vitro (release studies and cytotoxicity studies at 24 h and 72 h, *n* = 9)

**F**	**LC**	**TS**	**PS** **(nm)**	**PdI**	**ZP** **(mV)**	**DL %**	**R %**	C24* (µg/mL)	C72* (µg/mL)
SC KRH40	− 1 (SC)	− 1 (KRH40)	74 ± 2	0.181	− 27 ± 1	4.2 ± 0.1	25 ± 3	539 ± 59	415 ± 83
SC T80	− 1 (SC)	+ 1 (T80)	113 ± 2	0.258	− 26 ± 1	5.3 ± 0.4	29 ± 1	731 ± 83	578 ± 42
SSC KRH40	+ 1 (SSC)	− 1 (KRH40)	52 ± 2	0.195	− 24 ± 1	3.7 ± 1	21 ± 1	860 ± 52	746 ± 13
SSC T80	+ 1 (SSC)	+ 1 (T80)	121 ± 2	0.205	− 26 ± 1	3.6 ± 0.3	22 ± 1	639 ± 119	411 ± 52

*(in relation to lipid content)

The results are expressed as mean ± SD

Key: *F*, formulations; *LC*, lipid composition; *TS*, type of surfactant; *C*, Capryol™ PGMC; *S*, Suppocire^®^ NB; *SS*, Softisan^®^ 601 + Suppocire^®^ NB; *PS*, particle size; *PdI*, polydispersity index; *ZP*, zeta potential; *DL*, drug loading; *R*, release at 72 h; *C24*, cytotoxicity at 24 h (µg/mL); *C72*, cytotoxicity at 72 h (µg/mL)

**Fig. 6:**
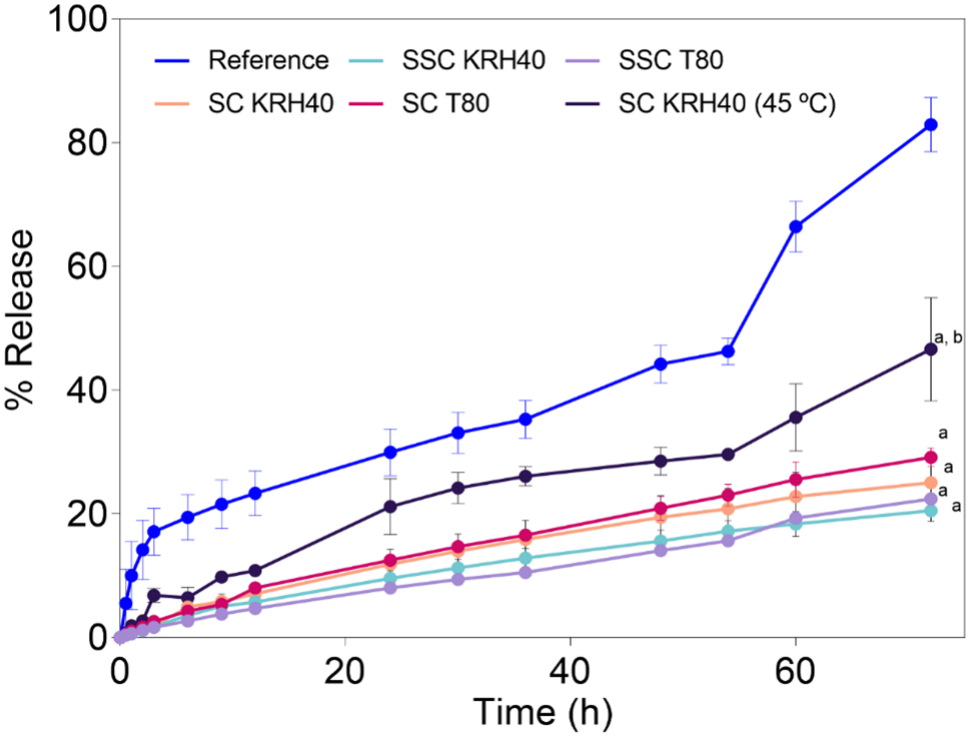
In vitro release profiles of CXB-usNLCs formulations reflecting the surfactants’ effect (Tween^®^ 80, T80; and Kolliphor^®^ RH40, KRH40) and lipid composition. Results are expressed as mean ± SD (*n* = 6). ^a^ CXB-loaded usNLCs formulations vs. reference, *p* < 0.01; ^b^ CXB-loaded SC KRH40 (45 °C) formulation vs. CXB-loaded usNLCs formulations*/*reference. Key: S:C, Suppocire^®^ NB:Capryol™ PGMC and S:S:C, Softisan^®^ 601: Suppocire^®^ NB:Capryol™ PGMC; T80, Tween^®^ 80; KRH40, Kolliphor^®^ RH40

The release profiles of CXB, which evaluated different usNLCs compositions, are presented in Fig. 6. Release studies allow obtaining preliminary information on the drug formulation behavior under the physiological conditions after systemic administration. The usNLCs formulations displayed similar release profiles, irrespective of the lipid composition, and showed a controlled drug release during the 72 h. Also, the usNLCs formulations provided a higher control over release of CXB than the reference (free CXB, soluble in Capryol™ PMGC, used as a vehicle, 83 ± 4%). The release profiles showed no correlation with the type of surfactant. The lipid combination SSC led to a lower release than SC, 22 ± 1% vs*.* 29 ± 1 (T80) and 21 ± 1% vs. 25 ± 3% (KRH40), respectively. The combination SSC (Suppocire^®^ NB:Softisan^®^ 601:Capryol™ PGMC) released a higher drug amount than SC (Suppocire^®^ NB: Capryol™ PGMC). However, the results did not show a statistically significant difference among the formulations. Interestingly, the amount of CXB released at 45 °C after 72 h was 47 ± 7%, corresponding to a double amount of drug released from the Suppocire^®^ NB: Capryol™ PGMC based formulation, when set at 37 °C (21 ± 1%, see Fig. 6). The incubation at 45 °C to simulate the hyperthermia condition induced the melting of the solid lipid core and allowed the CXB to partition from the melted lipid to the release medium, increasing its concentration throughout time. Such thermo-sensitive behavior could pave the way for a site-specific drug delivery when nanoparticles are laser irradiated in the target tissue. Note, however, that the in vivo effectiveness of this collective heating effect may depend not only on the temperature attained but also on the NP amount, and the respective biodistribution and conducting parameters of the matrix in which they are entrapped.

In terms of release kinetics, different regimes seem to be present in the same profile over the 72 h, which hampers the fitting of a sole mathematical model. As an example, the “reference” exhibited a burst release pattern during the first 3 h, followed by a sustained release until 56 h, and finally, in the last time points, a zero-order release. On the other hand, all the nanoparticles displayed a slow release and approach the characteristics of a Fickian diffusion mechanism, attaining around 7% of release after 12 h, with exception for SC KRH40 (45 °C) that showed a higher release after 12 h, further increasing the CXB released until the end of the study. Again, it reveals the impact of temperature on the kinetics of CXB release, sustaining the premise of the thermo-sensitivity of the nanoparticles.

**Fig. 7:**
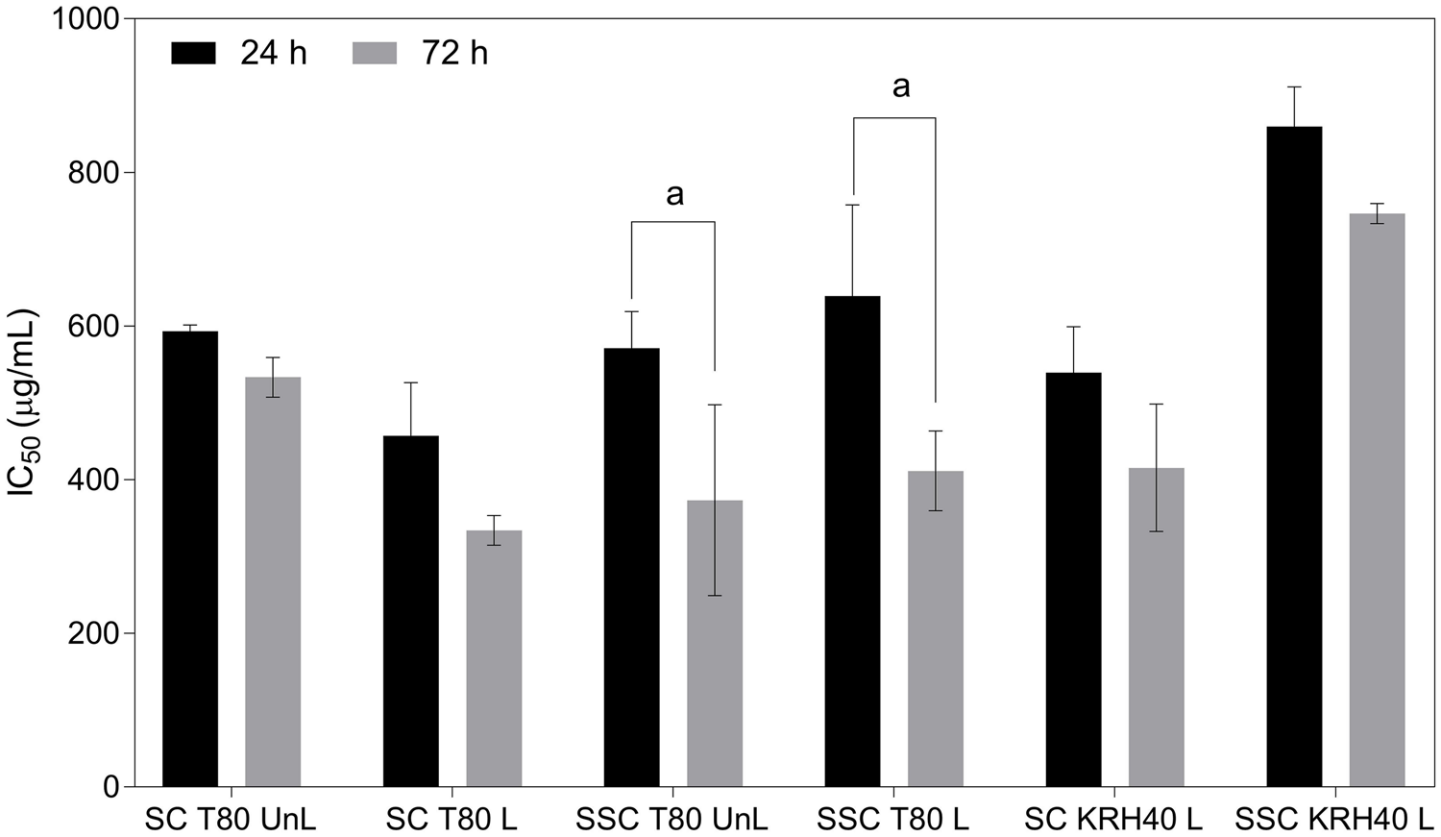
In vitro cytotoxic effect, as IC_50_, of different unloaded and CXB-loaded usNLCs incubated with U87 cells for 24 h and 72 h. Data are expressed as mean ± SD (*n* = 9, ^a^ *p* < 0.0001). Key: SC: Suppocire^®^ NB:Capryol™ PGMC, SSC: Softisan^®^ 601: Suppocire^®^ NB:Capryol™ PGMC; T80: Tween^®^ 80, KRH40: Kolliphor^®^ RH40; UnL, unloaded; L, loaded

Next, the potential cytotoxicity of the unloaded- and loaded-usNLCs was tested in GB cells. Although usNLCs have good biocompatibility, it is expected that at higher concentrations, the release of CXB contributes to cell growth inhibition. Different usNLCs, reported in Table 7, were suitably dispersed in DMEM and the respective cytotoxicity evaluated at 24 and 72 h. As explained before, usNLCs have different liquid:solid lipid combinations (Capryol™ PGMC + Softisan^®^ 601 + Suppocire NB, SSC; and Capryol™ PGMC + Suppocire NB, SC), and surfactants (Tween^®^ 80, T80, and Kolliphor^®^ RH40, KRH40). Both parameters were evaluated in terms of cellular viability (Fig. 7). The concentration of usNLCs added to cells was calculated considering the lipid content (0–4600 µg/mL). Cell viability was found to decrease with increasing duration of exposure to lipid nanoparticles, 24 to 72 h. CXB-loaded usNLCs have higher cytotoxicity than unloaded usNLCs. Unloaded SC KRH40 did not show cytotoxicity in the range of concentrations tested. Focusing on the surfactant, KRH40 was less cytotoxic than T80; considering the lipid composition, SC displayed a higher cytotoxic effect than SSC.

The design of experiments allows systematic control of process factors according to a predefined structure, improves process understanding, and determines optimal process conditions. Assessing the influence of CQAs on the final system, PS, PdI, ZP, DL, release, and cytotoxicity, as responses to experimental design enables an efficient control of predefined process factors.

To determine the influence of each CMA and the respective interaction, the polynomial coefficients were determined and are summarized in Table 8. A higher coefficient magnitude represents CMAs with a greater impact on the CQAs, while a negative coefficient bears the opposite trend. The analysis of the models shows better goodness of fit for particle size, cytotoxicity at 72 h, drug loading, and cytotoxicity at 24 h. In contrast, PdI and zeta potential displayed the worst goodness fit. These results are consistent with Fig. 6, which compares the experimental vs. predicted values for the CQAs. These curves provide a visual indication of whether the interest test is significant at the 5% level. If the confidence region between the curves contains the horizontal line representing the hypothesis, the effect is irrelevant. The result is substantial if the curves cross the line, as observed for most responses.

**Table 8:** Values of coefficients for particle size (PS), polydispersity index (PdI), zeta potential (ZP), drug loading (DL), percentage of CXB released at 72 h (%R), cytotoxicity in U87 at 24 h (C24), and 72 h (C72), and respective summary of fit of the selected critical material attributes. *Statistically significant coefficients, as extracted from Student’s t-test analysis

	**PS**	**Prob >|*t*|**	**PdI**	**Prob >|*t*|**	**ZP**	**Prob >|*t*|**	**DL**	**Prob >|*t*|**
***β***_**0**_	90.270	< 0.0001*	0.209	< 0.0001*	− 25.756	< 0.0001*	4.191	< 0.0001*
**LC**	3.675	0.0004*	0.010	0.5065	− 0.744	0.0305*	0.541	0.0024*
***S***	− 26.813	< 0.0001*	− 0.022	0.1616	0.256	0.3943	− 0.238	0.0906
LC*S	7.291	< 0.0001*	− 0.017	0.2675	− 0.622	0.0597	− 0.299	0.0422*
***R***^**2**^	0.996		0.350		0.610		0.780	

	**%R (72 h)**	**Prob >|*t*|**	**C24**	**Prob >|*t*|**	**C72**	**Prob >|*t*|**		
***β***_**0**_	26.641	< 0.0001*	692.258	< 0.0001*	537.890	< 0.0001*		
**LC**	1.186	0.4377	− 57.108	0.0431*	− 41.008	0.0292*		
***S***	0.862	0.5694	7.242	0.7685	42.958	0.0240*		
LC*S	− 6.6040	0.0019*	− 103.058	0.0025*	− 124.475	< 0.0001*		
***R***^**2**^	0.730		0.750		0.910			

**Fig. 8:**
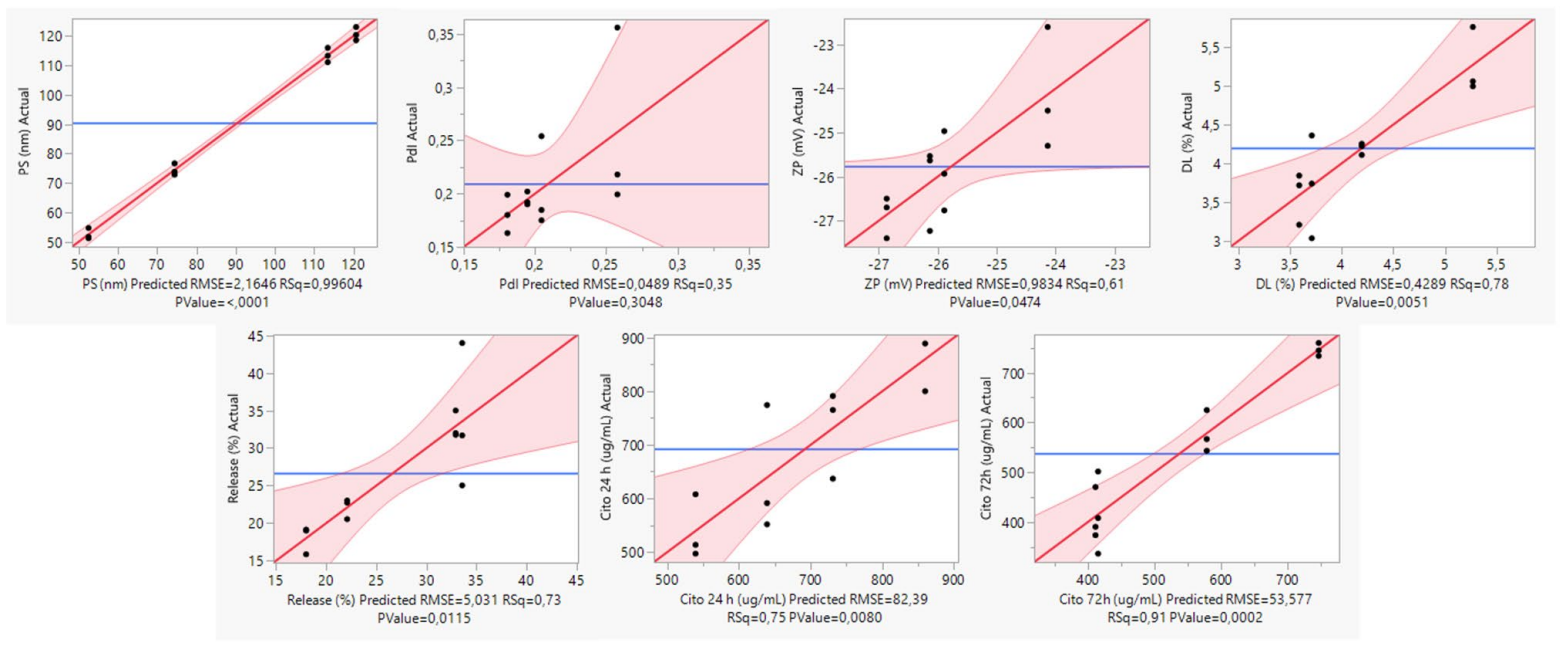
Actual by predicted plots for the responses (CQAs) presenting better goodness of fit. The diagonal line corresponds to the *Y* = *X* line. For a perfect fit, all the points would be on this diagonal. The horizontal line indicates the meaning of each response (*Y*-residuals). Confidence curves for the line of fit are shown on leverage plots

The study of the effect on the usNLCs colloidal properties showed that the reduction of particle size depends on the type of surfactant (KRH40 vs. T80), with KRH40 having a high relevance to the reduction in particle size, followed by the interaction between the lipid composition and the surfactant, SC KRH40. On the other hand, DL was influenced by the lipid composition, and to a lesser extent by the surfactant. Interestingly, an increase in the amount of Suppocire^®^ NB on the lipid matrix resulted in a higher drug release, see Fig. 8. This can be confirmed by the low melting point of the solid lipid (~ 38 °C). In cytotoxicity studies, at 24 h there was a higher impact of lipid composition; at 72 h, the interaction between CMAs had an increased effect on cytotoxicity, resulting in a higher cytotoxicity of SC KRH40.

**Fig. 9:**
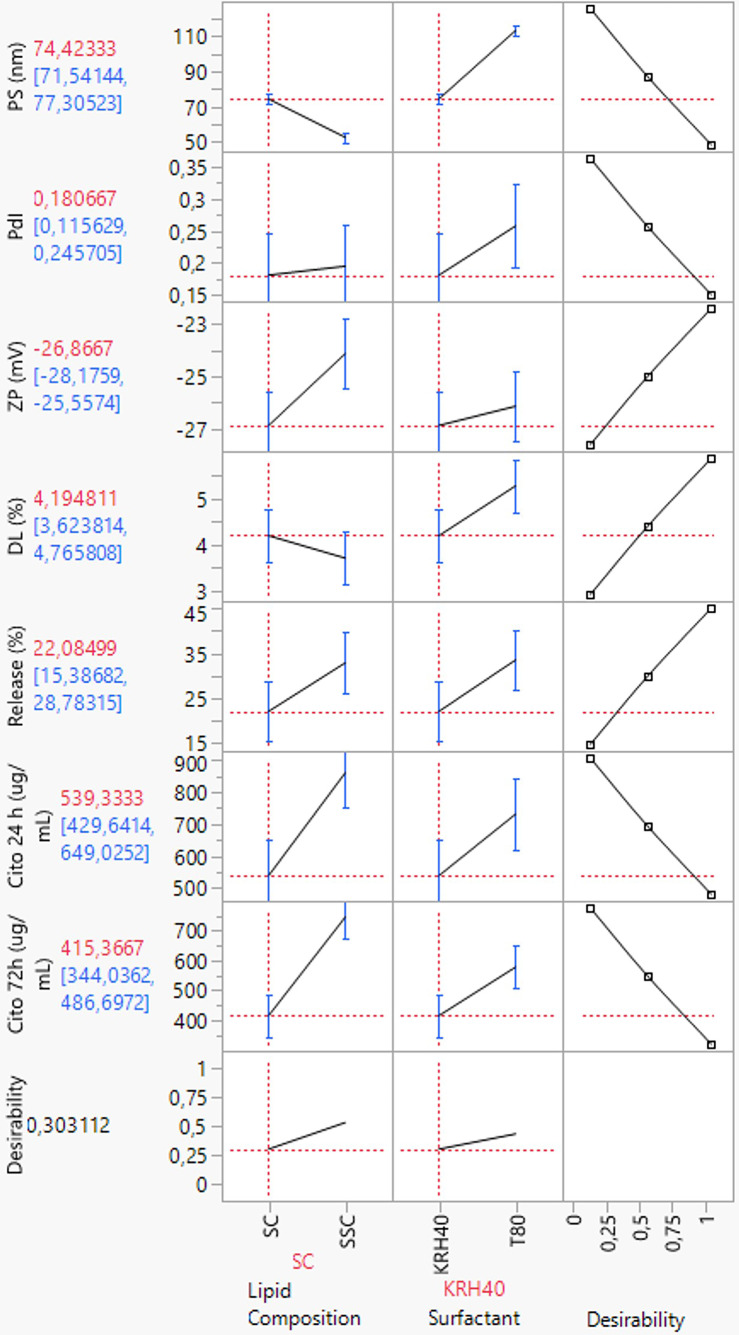
Overall desirability for usNLCs composition optimization, according to the target (increase or decrease) imposed per CQAs and formulation stability. The last row corresponds to the desirability trace combining all the factors. The overall desirability for responses was defined as the geometric mean of the functions for the individual response

Figure 9 shows the overall desirability function applied to the optimization of independent variables for desirable responses. The desirability function combines all responses that can be maximized or minimized according to the CMAs into one measurement and provide a way to predict the optimal values of independent variables [62, 63]. The desirability range is a value comprised between 0 and 1 per response. A higher desirability (value = 1) means that the combination of the different criteria is considered optimal. The formulation containing Kolliphor^®^ RH 40 as surfactant (level 1, KRH40) and only one solid lipid (level 1, SC) configures the effects of reducing PS, PdI, ZP, release rate, and IC_50_, while maximizing drug loading as optimal settings to ultimately obtain a stable formulation.

## Conclusions

The effectiveness of chemotherapy is limited in cancer treatment, including GB, due to overexpression of resistance mechanisms, lack of tumor selectivity, and severe side effects. TMZ is no exception. For these reasons, an advance in research considering drug repurposing provides hope to GB patients. The drug repurposed can be selected to target specific proteins and disrupt the molecular cascades that drive tumor progression, migration, or metastasis.

In the first part of the work, the strategy of “drug repurposing” was investigated considering bioinformatics tools and in vitro cellular studies to sustain the use of CXB as potential anticancer drug for GB treatment. Following this path, CXB was further evaluated and compared to the first-line treatment (TMZ) in four glioma cells. The results showed that CXB inhibited the glioma cells in a higher extension than TMZ. The combinatory effects of CXB and TMZ were measured, and no synergistic effect was observed between the two drugs (CI > 1). Therefore, only CXB was selected for subsequent studies. Despite the advantages of using CXB as a GB chemotherapeutic agent, its physicochemical properties need to be improved.

For this reason, the second part of the work provisioned the design of thermoresponsive usNLCs for the treatment of GB. To optimize the formulations, a QbD approach was defined based on FMECA analysis, considering critical material attributes with higher risk. The optimization studies based on experimental design led to the production of usNLCs with the desired pre-defined characteristics, which were supported on (i) small particle size (< 100 nm), (ii) high lipid concentration (15% w/w), (iii) high drug loading (5% w/w), and (iv) nanoparticle stability. Using a lipid matrix of Suppocire^®^ NB:Capryol™ PGMC stabilized by Kolliphor^®^ RH40, monodisperse usNLCs, with small particle size (*ca*. 70 nm), high stability, and drug loading was developed. usNLCs have proved to be an effective lipophilic drug carrier, without changes upon usNLCs properties. Additionally, in vitro cytotoxicity studies showed that CXB-usNLCs can fulfill the purpose of provisioning anticancer activity against GB cells.

Overall, nanoparticle design considering a combinatorial strategy that includes different variables, ranging from physicochemical characteristics of the drug to the in vitro performance, is a simple but effective approach that provides information to strongly support the best decision for formulation optimization. Thus, this work provides a thermoresponsive nanoparticle resorting to usNLCs that can be combined with external stimuli (*e.g.*, hybridized with gold nanoparticles activated by near-infrared radiation) to trigger controlled CXB release to the tumor.

## Data Availability

The raw data required to reproduce these findings are available on reasonable request from the corresponding author (C.V.).
